# Gap junctions desynchronize a neural circuit to stabilize insect flight

**DOI:** 10.1038/s41586-023-06099-0

**Published:** 2023-05-24

**Authors:** Silvan Hürkey, Nelson Niemeyer, Jan-Hendrik Schleimer, Stefanie Ryglewski, Susanne Schreiber, Carsten Duch

**Affiliations:** 1 Institute of Developmental Biology and Neurobiology (iDN), Johannes Gutenberg-University Mainz, Mainz, Germany; 2 Institute for Theoretical Biology, Humboldt-Universität zu Berlin, Berlin, Germany; 3 Bernstein Center for Computational Neuroscience Berlin, Berlin, Germany

**Keywords:** Central pattern generators, Motor neuron, Dynamical systems

## Abstract

Insect asynchronous flight is one of the most prevalent forms of animal locomotion used by more than 600,000 species. Despite profound insights into the motor patterns^1^, biomechanics^2,3^ and aerodynamics underlying asynchronous flight^4,5^, the architecture and function of the central-pattern-generating (CPG) neural network remain unclear. Here, on the basis of an experiment–theory approach including electrophysiology, optophysiology, *Drosophila* genetics and mathematical modelling, we identify a miniaturized circuit solution with unexpected properties. The CPG network consists of motoneurons interconnected by electrical synapses that, in contrast to doctrine, produce network activity splayed out in time instead of synchronized across neurons. Experimental and mathematical evidence support a generic mechanism for network desynchronization that relies on weak electrical synapses and specific excitability dynamics of the coupled neurons. In small networks, electrical synapses can synchronize or desynchronize network activity, depending on the neuron-intrinsic dynamics and ion channel composition. In the asynchronous flight CPG, this mechanism translates unpatterned premotor input into stereotyped neuronal firing with fixed sequences of cell activation that ensure stable wingbeat power and, as we show, is conserved across multiple species. Our findings prove a wider functional versatility of electrical synapses in the dynamic control of neural circuits and highlight the relevance of detecting electrical synapses in connectomics.

## Abstract (web summary)

In the *Drosophila* central-pattern-generating neural network, a mechanism for network desynchronization relying on weak electrical synapses and specific excitability dynamics of the coupled neurons translates unpatterned premotor input into stereotyped neuronal firing with fixed sequences of cell activation, ensuring stable wingbeat power.

## Main

With over a million known species, insects comprise the largest group of animals on Earth^6^. Their considerable evolutionary success has been attributed to small body size and the ability to fly. These two features provide access to unutilized niches and rapid translocation, but aerodynamic constraints in small flyers require high wingbeat frequencies, and space constraints demand miniaturization of the central nervous controllers for flight^7^. In 75% of all flying insect species, highly specialized, indirect, asynchronous flight muscles form an oscillatory system that generates wingbeat frequencies of 100–1,000 Hz by reciprocal stretch activation of antagonistic wing muscles to ensure forward propulsion at low Reynolds numbers^1,8^. The flight motoneurons (MNs) that innervate asynchronous flight muscles fire at much lower frequencies, therefore not activating the muscles on a cycle-to-cycle basis. Nonetheless, power output is regulated by a CPG network in the central nervous system that controls MN firing frequencies to adjust the myoplasmic calcium levels that, in turn, regulate wingbeat frequency and amplitude^1^. Although asynchronous flight has emerged independently 7–10 times during evolution^8^, neither the principles of CPG architecture for generating MN output from the miniaturized central nervous system of asynchronous flyers nor the functional consequences thereof have been identified.

## Asynchronous flight motor patterns

**Fig. 1:**
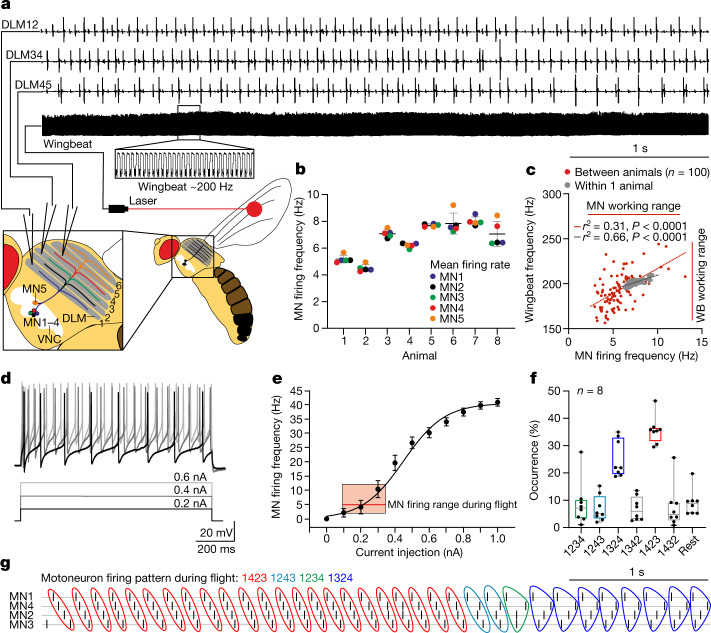
Splayed-out MN firing patterns control *Drosophila* flight. **a**, Representative recording of MN1–5 and wingbeat frequency (bottom trace, magnified in the black box) during tethered flight. Colour-coded schematic of MN1–5 in the VNC and axonal projections to the six fibres of the DLM. **b**, Average MN firing frequencies are similar within each animal. The colour code is the same as in **a**. *n* = 8 animals. Data are mean ± s.d. **c**, MN firing frequency and wingbeat (WB) frequency (the red bars indicate working ranges) are linearly related within an animal (grey dots; correlation coefficient, *r*^2^ = 0.63; *P* < 0.0001, two-tailed *t*-test) and with larger variance also across animals (red dots; *n* = 100; correlation coefficient, *r*^2^ = 0.31; *P* < 0.0001, two-tailed *t*-test). **d**, The firing responses of MNs (top traces) to current injections of different amplitudes (bottom traces). **e**, The mean MN response frequency (*f*) and injected current amplitude (*I*) are approximately linearly related for 2–30 Hz (*n* = 15 animals), therefore exceeding the normal MN firing frequencies observed during flight (inset; ~3–12 Hz, data from **c**). Data are mean ± s.e.m. (main plot) and median ± range (inset). **f**, During flight, MN1–4 spikes are dispersed in time (in splay state) with characteristic sequences. Each animal switches between different splay states during flight, but the same splay states are preferred across individuals (*n* = 8). The box plots show the median (centre line), quartiles (box limits) and range (error bars). **g**, Timing of MN1–4 spikes in four subsequent splay states (1423 (red); 1243 (turquoise); 1234 (green); 1324 (dark blue)) during flight.

To quantify asynchronous flight patterns and decipher CPG architecture, we used the firing output of the five identified MNs (MN1–5) innervating the dorsal longitudinal wing depressor muscle (DLM) of the genetic model system^9–11^ *Drosophila melanogaster* as well as other insect species (to test for generality). The DLM provides the force for wing downstroke, consists of six muscle fibres, each of which is innervated by one of five identified MNs^9–11^ (MN1–5; Fig. 1a). MN1–4 each target one of the four most ventral DLM fibres ipsilateral to their somata, whereas MN5 innervates DLM fibres 5 and 6 on the side contralateral to the MN5 soma (Fig. 1a). This neuromuscular architecture is conserved across insect species examined (locust^12^, moth^13^, blowfly^14^).

In vivo recordings of MN1–5 from their DLM target muscle fibres using simultaneous laser-based wingbeat detection during tethered flight show that each MN fires only every approximately 20th to 40th wingbeat (Fig. 1a–c). Although the firing frequencies of MN1–5 can vary between animals and are adjusted on demand^1,15,16^, within a given animal and power demand, all five MNs always fire at the same frequencies (Fig. 1b) and with similar variance in the interspike interval (ISI; coefficient of variation is 0.22 ± 0.07 (mean ± s.d.) for each MN).

MN1–5 firing frequencies directly control muscular tension and stretch activatability by adjusting myoplasmic calcium levels and, therefore, wingbeat frequency and stroke amplitude^1,17^. Accordingly, in single animals, alterations in power demands go along with changes in MN1–5 firing frequency that are proportional to wingbeat frequency changes (Fig. 1c (grey dots)). A linear relationship between average MN firing and average wingbeat frequency within the normal flight working range was further confirmed by recordings in 100 animals, although interanimal variability yields a lower correlation between MN firing rate and wingbeat frequency (Fig. 1c (red dots)). This correlation is increased when analysing the changes in the MN firing rate in relation to changes in the wingbeat frequency (Extended Data Fig. 4e–g). Thus, the central nervous system controls asynchronous flight muscle power output neither by the recruitment of different motor units, nor on the scale of single wingbeats, but the frequency of MN1–5 population firing is the key regulator of wing power production^1^. We show that MN excitability is well suited to dynamically regulate wing power output. First, MN1–5 respond to constant input with slow tonic firing (Fig. 1d). Second, a nearly linear input–output relationship as observed in *f*–*I* curves (3–30 Hz; Fig. 1e) covers and even exceeds the working range observed during tethered flight (approximately 3–12 Hz; Fig. 1c). The excitability of MN1–5 is therefore tuned to linearly translate synaptic input into tonic firing to regulate wingbeat in the working range of flight.

**Extended Data Fig. 1:**
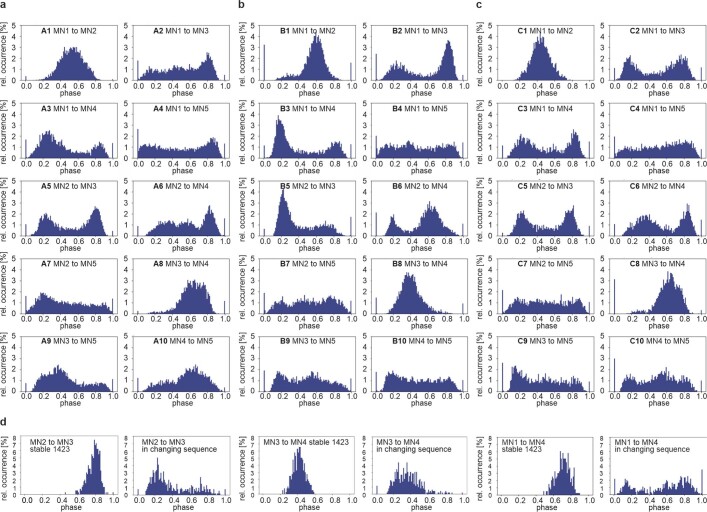
Firing phase relationships of MN1–5 pairs are conserved between individuals. For three individual male flies **(a–c)** all 10 possible pairwise combinations of the DLM-MNs (MN1–5) are plotted as cumulative phase histograms from simultaneous recordings of MN1–5 during 10 min of continuous tethered flight (equalling approximately 3000 spikes of each MN). Starting with the MN1/MN2 pair on the upper left, **(a1-c1)**, for each MN pair, the interspike intervals between two consecutive spikes of one MN were divided into 100 equally sized bins (x-axis), and it was determined in which bin the spike of the other MN occurred. This was repeated as sliding window for all interspike intervals to fill the bins cumulatively. Cumulative spike counts in each bin were normalized to total spike count (y-axis). In all three individuals **(a–c)** different MN pairs show different phase relationships, but the same pairs show similar phase relationships across individuals. The phase histograms of all MN pairs show a characteristic gap around phases zero and 1, indicating preferred out-of-phase firing. Apart from this characteristic gap, phase relations are not highly precise, so that some distributions are broad. This is owed to multiple preferred MN1–5 firing sequences (splay states) occurring within one flight bout (see Fig. 1 in the main text). **(d)** compares phase histograms restricted to one splay state and with changing splay states (both from the same animal and 1 min of flight). Histograms restricted to one splay state show a much narrower phase relation and no synchronous spikes at phases 0 or 1.

Despite equal firing frequencies at any given wingbeat frequency^1,15^ (Fig. 1a,b), the five MNs in this system do not fire in synchrony. Instead, as previously suggested^18^, their spikes are splayed-out in time with firing phases across neurons dispersed approximately equidistantly, resulting in stereotyped preferred sequences of MN1–5 spiking (Fig. 1g), which we name splay states. A network splay state means that each individual cell in a network of *N* neurons fires regularly, yet with a constant, non-zero phase relationship with respect to the firing of each other neuron. Firing phase differences between neurons approximately correspond to 1/*N*th of a neuron’s ISI or a multiple (<*N*) thereof. Importantly, the sequence of MN1–5 firing can change intermittently, but the CPG robustly slides back into one or two of the most preferred splay states. Notably, the same splay states are preferred across animals (Fig. 1f), indicating that they result from hard-wired CPG circuitry. Splayed-out firing results in characteristic phase relationships between each pair of MN1–5. These phase relationships are conserved across individuals (Extended Data Fig. 1a–c). The phase relationships of all MN1–5 pairs show a gap around phase zero, indicating out-of-phase firing. Given that multiple splay states exist during flight (Fig. 1f,g), firing of MN pairs is not precisely phase locked (Extended Data Fig. 1a–c). Yet, restricting phase histograms to one splay state narrows the phase relations (Extended Data Fig. 1d). Moreover, synchronous spikes between MN pairs occasionally occur during switches between different splay states, but not within a stable splay state (Extended Data Fig. 1d).

**Extended Data Fig. 2:**
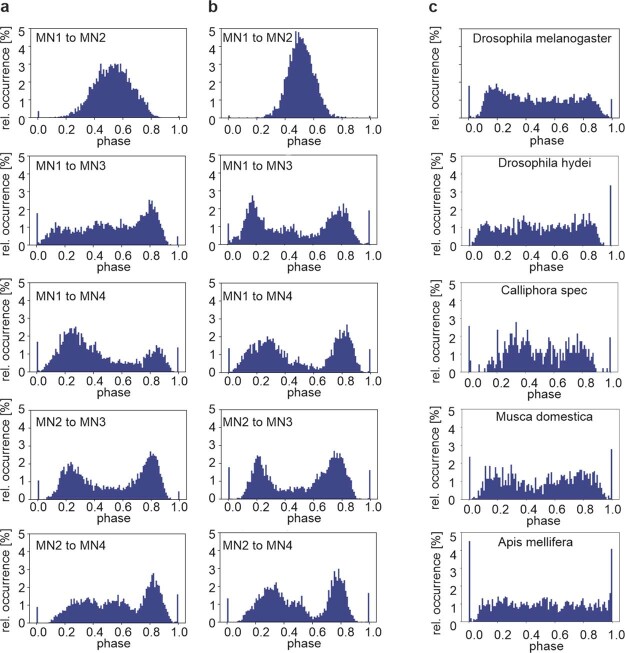
Phase relations between MN1–5 are conserved across species. In all tested flying insect species, the dorsal longitudinal wing depressor muscles (DLM) consists of 6 muscle fibres, each of which is innervated by one MN, namely MN1–5 (for schematic see Fig. 1a). In *Drosophila melanogaster* each of the 10 MN1–5 pairs shows characteristic phase relationships in their firing patterns (see Fig. 1 in the main text, Extended Data Fig. 1). Analyses of *in vivo* recordings during tethered flight reveals highly similar firing phase relationships of different MN pairs in *Drosophila melanogaster* **(a)** and in the goldfly, *Lucilia spec* **(b)**, a related dipteran species. **(c)** *In vivo* recordings of the MN4-MN5 pair shows similar firing phase relationships between the fruit fly *Drosophila melanogaster* (top), another Drosophilidae (*Drosophila hydei*, second from top), two additional dipteran species (the blowfly *Calliphora spec*., third from top; and the house fly, *Musca domestica*, second from bottom). Some characteristics of the phase relationships observed in all dipteran species tested (top 4 panels) are also observed in a hymenopteran species, the honey bee *Apis mellifera* (bottom). There the characteristic inhibition of MN4 firing just after MN5 spikes is recapitulated, but the depression of MN4 firing just before MN5 spikes is not.

Our characterization of phase relationships between pairwise combinations of the MN1–5 enables us to test for across-species conservation of the motor patterns (see Supplementary Videos 1–3 for the flight of three species). This reveals a notable similarity between *D. melanogaster* and the gold fly *Lucilia* sp. (Extended Data Fig. 2a,b). Similar phase relationships have also been suggested for *Calliphora erythrocephala*^14^, *Eucaliphora lilaea* and *Musca domestica*. Moreover, using the MN4–MN5 pair (Extended Data Fig. 2c), our measurements indicate conservation of CPG architecture between two *Drosophila* species (*D. melanogaster* and *Drosophila hydei*), other dipteran genera (*Calliphora* and *Musca*) and, to a certain degree, even between *Diptera* (for example, flies) and *Hymenoptera* (for example, honey bee). Given that asynchronous flight evolved multiple times independently^8^, splayed-out firing has probably provided selective benefits over millions of years. To gain mechanistic and functional insights into the CPG network that controls asynchronous flight, we next analysed the network principles underlying splay state motor pattern generation and the resulting functional benefits for flight performance.

## Network splay states produced by a minimal CPG

**Extended Data Fig. 3:**
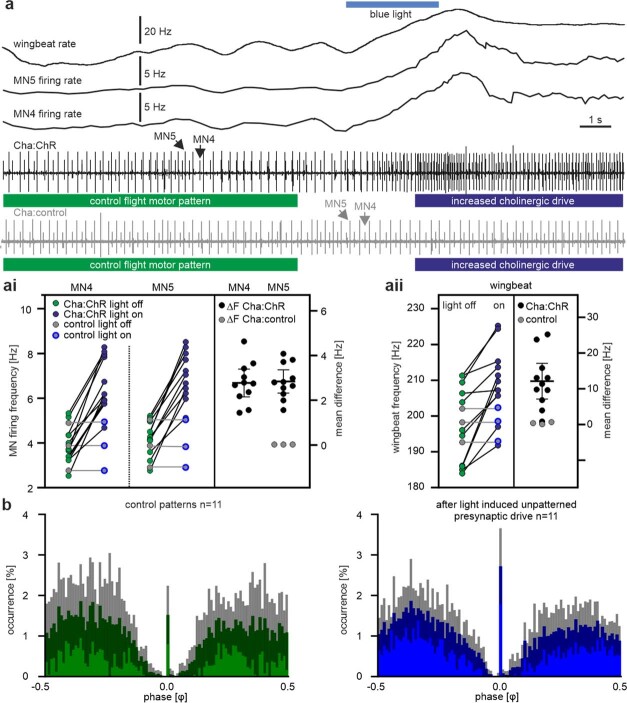
Unpatterned excitatory input scales up CPG output without affecting the pattern. **(a)** Representative recordings of MN4 (arrow, smaller unit) and MN5 (arrow, large unit) with one tungsten electrode during tethered flight and blue light stimulation in a fly with expression of UAS-Channelrhodopsin in cholinergic neurons (Chat-GAL4xUAS-XXL-ChR, second from bottom; 11 replicate animals) and in a control fly (Chat-GAL4x w^1118^, bottom trace; 3 replicate animals). Blue bars indicate the timing of blue light stimulation. Upper three traces show the instantaneous wingbeat frequency (top trace) and the instantaneous firing frequencies of MN5 and MN4 upon optogenetic activation of presynaptic cholinergic neurons. **(ai, left)** Quantification from 11 animals with expression of UAS-ChR in cholinergic neurons reveals that the firing frequencies of MN4 and MN5 are increased significantly and to the same degree during optogenetic stimulation (blue circles) as compared to before light stimulation (green circles). By contrast, in controls (Chat-GAL4 x w1118; n = 3) light stimulation has no effect. **(ai, right)** Average firing frequency increases in MN4 and MN5 (black dots) but not in controls (grey dots, horizontal bars depict means, error bars the SDs). **(aii)** Consequently, wingbeat frequency is significantly increased upon optogenetic activation of cholinergic neurons (11 animals, horizontal bar depicts mean, error bars the SD), but not upon light stimulation in controls (3 animals). **(b)** The typical phase relationship of MN4 and MN5 firing that is observed in controls (Fig. 2a,b in the main text, Extended Data Fig. 1) and prior to optogenetic stimulation (left) remains unaltered upon increasing CPG output by unpatterned stimulation of presynaptic cholinergic interneurons (right). Coloured bars represent the average values from 11 animals and grey bars the SD.

**Extended Data Fig. 4:**
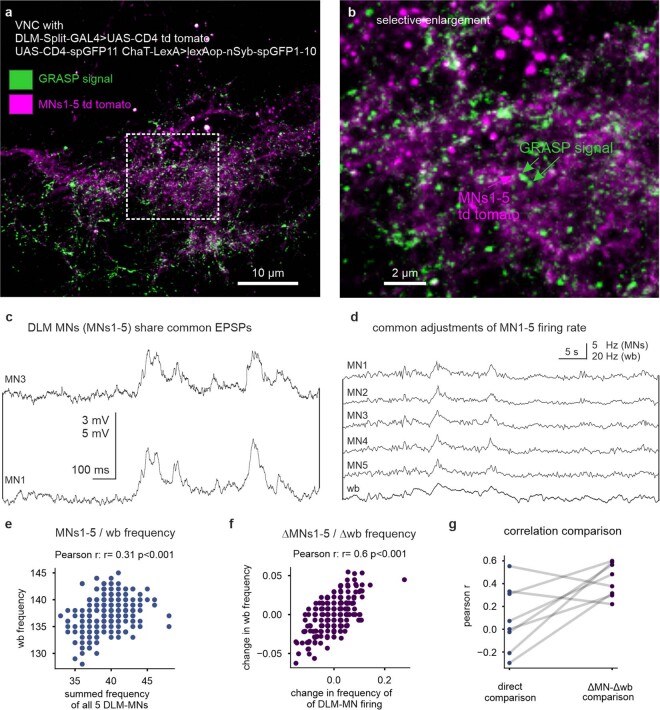
MN1–5 receive common excitatory cholinergic input and control wingbeat rate. **(a)** Representative confocal projection view (5 replicate animals) of the VNC flight motor neuropil in an animal with expression of UAS-CD4 td tomato (magenta) and one half of split-GFP (UAS-CD4-spGFP11) under the control of GAL4 in DLM-MNs (driven with GMRH2306-ADZ attP40; GMR30A07-DBD attP2, see also Extended Data Fig. 5a,b for expression patterns with UAS-GFP). The other half of split-GFP (spGFP1-10) is coupled to the synaptic vesicle protein synaptobrevin (nSyb) and driven under the control of LexA in cholinergic neurons (ChaT-LexA x lexAop-nSyb-spGFP1-10). GFP reconstitution across synaptic partners (GRASP) occurs upon release of synaptic vesicles from cholinergic neurons and results in GFP fluorescence at the sites where nSyb- spGFP1-10 is released in direct proximity of MN1–5 dendritic membrane with expression of CD4-spGFP11 (green). **(b)** Selective enlargement of synaptic activity dependent GRASP signal (green) at contacts between ChaT positive cholinergic neurons and td tomato expressing MN dendrites (magenta) indicates direct synaptic inputs from cholinergic neurons to MN1–5. **(c)** Dual current clamp recording from 2 MNs (here MN3 and MN4) show highly similar patterns of PSPs, providing direct evidence for shared synaptic input (representative traces from 1 animal, 4 replicate animals). **(d)** Representative example for similar changes in firing frequency of MN1–5 indicates shared synaptic drive (8 replicate animals). **(e)** Wingbeat frequencies and MN1–5 firing frequencies are calculated for 1-second bins within flight. Flight starts and stops are omitted to avoid trivial correlations. The correlation between MN1–5 firing frequency and wingbeat frequency is moderate across animals, though significant (two-sided Pearson correlation coefficient r = 0.31, p < 0.0001). **(f)** In the same animal, changes in MN1–5 (ΔMN) and wingbeat frequency (Δwb, comparing consecutive 1-second bins) show a stronger correlation (Pearson r = 0.6, p < 0.0001). **(g)** The ΔMN/Δwb correlation is also stronger across animals.

**Extended Data Fig. 5:**
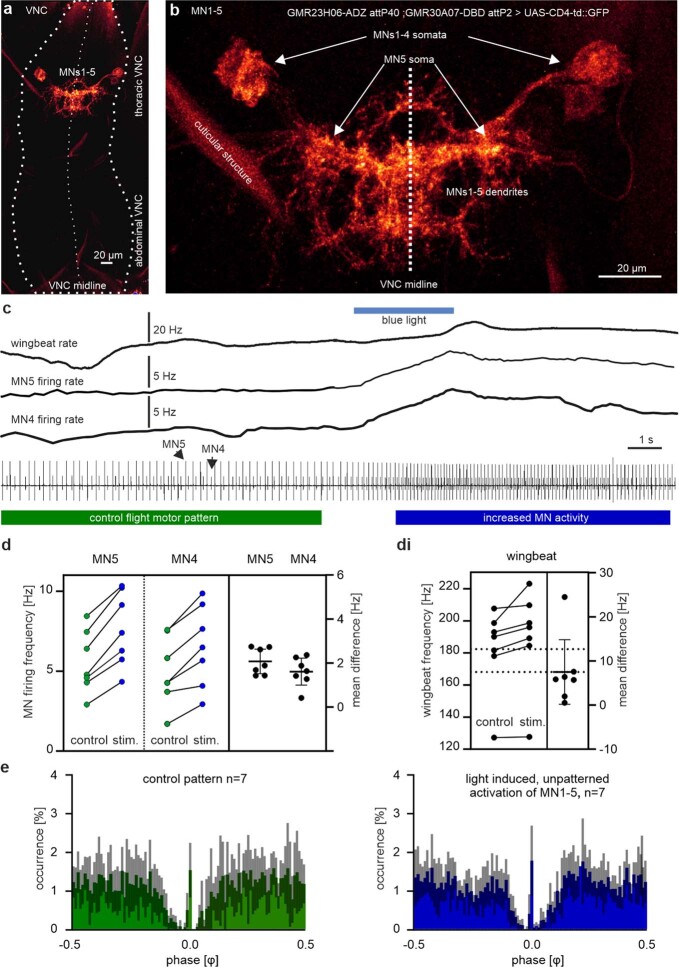
Unpatterned optogenetic activation of MN1–5 during flight scales up CPG output without affecting the pattern. **(a)** Representative projection views of confocal image stacks (5 replicate animals) as overview of the entire ventral nerve cord (VNC, outlined by dotted line) and **(b)** as selective enlargement of the pterothoracic VNC with a expression of UAS-CD4-td::GFP under the control of DLM MN Split-GAL4 (GMR23H06-ADZ attP49; GMR30A07-DBD attP2). The VNC midline is indicated by a dotted white line, MN1-4 somata are labelled by white arrows on both sides of the VNC. MN5 somata (white arrows) are located closer to the midline and partially obscured by MN1–5 dendrites in the flight motor neuropil. The only other signals detected are due to autofluorescence from cuticular structures. **(c,d)** Optogenetic activation of MN1–5 during flight. **(c)** The bottom trace shows a representative recording of MN4 (arrow, smaller unit) and MN5 (arrow, large unit) with one tungsten electrode during tethered flight. Timing of unpatterned optogenetic activation of MN1–5 is indicated by a blue line. Upper three traces show the instantaneous wingbeat frequency (top trace) and the instantaneous firing frequencies of MN5 and MN4 (7 replicate animals). **(d)** Quantification shows that optogenetic activation increases the firing frequencies of both MNs significantly and to the same degree (horizontal bar depicts mean, error bars the SD). **(di)** Consequently, wingbeat frequency is increased significantly (n = 7 animals, horizontal bar depicts mean, error bars the SD). **(e)** The typical phase relationship of MN4 and MN5 firing that is observed in controls (Fig. 2a, Extended Data Fig. 1) and prior to optogenetic stimulation (left) remains unaltered upon increasing CPG output by unpatterned stimulation the MN1–5 (right). Coloured bars represent the average values from 7 animals and grey bars the s.e.m.

**Extended Data Fig. 6:**
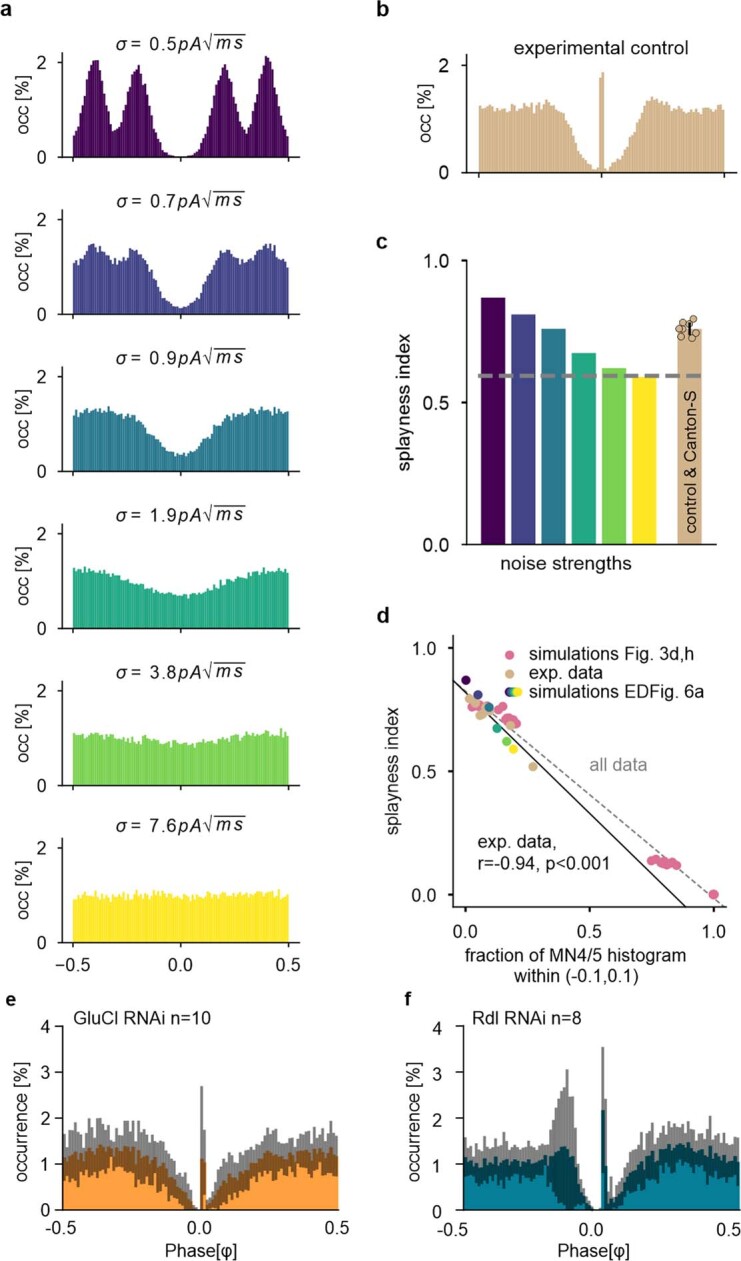
Dip around zero in phase histograms is an appropriate indicator for splayness and not affected by inhibitory chemical synapses. **(a**) Phase histograms for simulated MN1–5 with homogeneous coupling (occ [%] is the percentage of events in each phase bin). All-to-all rather than single-pair histograms are chosen to mitigate initial condition effects for finite run times. For low noise 4 peaks correspond to the 4 phase relations in a perfect splay state. With increasing noise, the dip around zero is more persistent than the subsequent dips. **(b)** Similar all-to-all phase histogram for an example experimental control. **(c)** Washing out of the dip around zero corresponds to decreased network splayness (y-axis, splayness index). For comparison, mean and standard deviation of experimentally measured control and Canton-S flies are shown (n = 8 animals). **(d)** For MN4-5 histograms the dip around zero is quantified using the fraction that falls within (−0.1,0.1). This fraction is negatively correlated to full network splayness in both simulated (considering all simulations from Fig. 3d,h) and experimental data where all 5 neurons were available (Canton-S, experimental control (GMR23H06-ADZ attP49;GMR30A07-DBD attP2 > P{UAS-GFP.VALIUM10}attP2), ShakB RNAi-kd (GMR23H06-ADZ attP49;GMR30A07-DBD attP2 > P{TRiP.HMC04895}attP2)). For the experimental data, a two-sided Pearson correlation determined a strong negative correlation (r = −0.94, p < 0.0001, n = 10). **(e,f)** Firing phase relationships between MN4 and MN5 in control animals (see also Fig. 2a,b, Extended Data Fig. 1) remain similar upon UAS-RNAi knockdown of receptors for inhibitory chemical synapses in MN1–5 (under the control of DLM-MN spilt-GAL4, *GMR23H06-ADZ attP49; GMR30A07-DBD attP2*), **(e)** the glutamate gated chloride channel (GluCl) and **(f)** Rdl GABA-ARs. GluCl-RNAi knockdown efficacy was confirmed by Western blotting and Rdl GABA-AR knockdown efficacy has previously been confirmed^64^. Coloured bars represent the average values from 10 animals for GluCl-RNAi, 8 animals for Rdl GABA-AR-RNAi, and grey bars the s.e.m.

**Extended Data Fig. 7:**
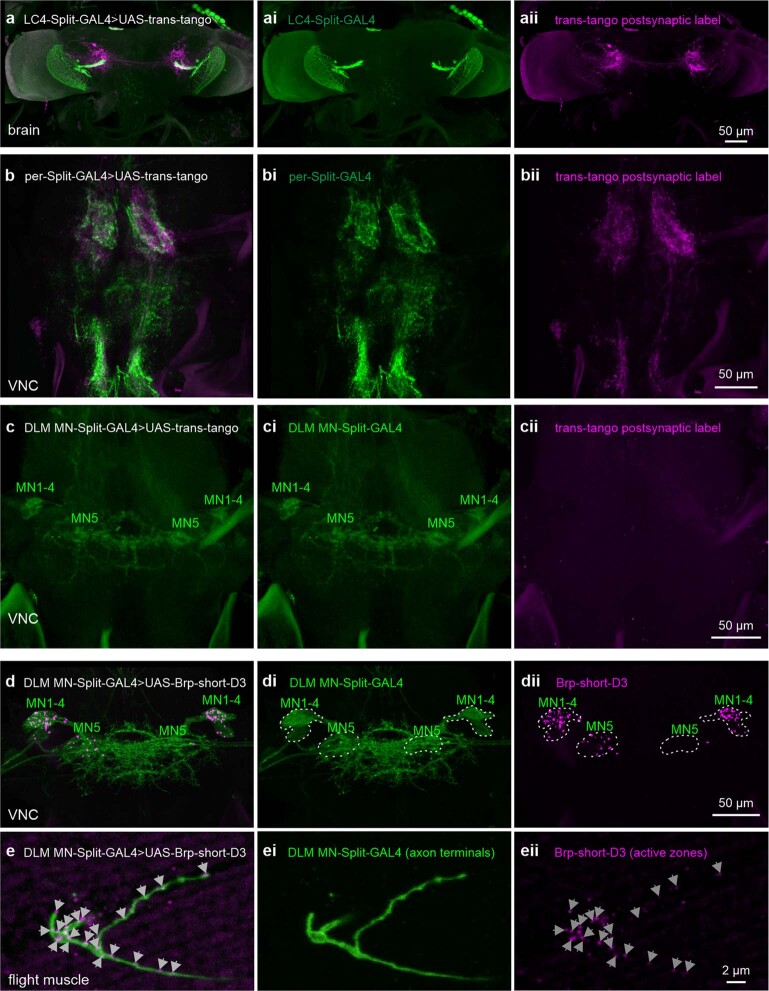
No detection of output synapses from MN1–5 in the ventral nerve cord (VNC). **(a)** Representative (from 4 replicate animals) trans-synaptic labelling of postsynaptic partners (**a, aii** magenta) of LC visual interneurons (**a, ai**, green) in the adult fly brain with Trans-Tango^65^. **(b)** Representative (from 4 replicate animals) trans-synaptic labelling of postsynaptic partners (**b, bii** magenta) of period interneurons in the adult VNC (**b, bi**, green). **(c)** By contrast trans-synaptic labelling with Trans-Tango reveals no postsynaptic partners (**c, cii**, magenta) of MN1–5 (**c, ci**, green) in the adult fly VNC (6 replicate animals). **(d)** Expression of Brp-short (w;23H06-ADZ UAS-Brp.S^straw-^D3/+;30A07-DBD/+;) tagged with strawberry in MN1–5 (4 replicate animals) causes Brp-short strawberry puncta in the MN1–5 somata, where the protein is produced, but not in any central arbours (**d, di, dii**, MN1–5 green, Brp short magenta). By contrast, in the same preparation, Brp-short puncta (white arrows) localize to presynaptic active zones at MN axon terminals at the neuromuscular junction (**e, ei, eii**, MN1–5 green, Brp short magenta).

From invertebrates^19^ to mammals^20^, the timing and pattern of MN activation during locomotion typically rely on networks of premotor interneurons. By contrast, we provide evidence that splayed-out MN1–5 firing is generated by interactions between the MNs themselves. First, unpatterned optogenetic activation of excitatory, cholinergic input to MN1–5 increases MN firing frequencies without changing their phase relationships, preserving a characteristic gap around phase 0 (Extended Data Fig. 3). We confirmed both physiologically^21^ and anatomically that MNs receive excitatory cholinergic input to their dendrites (Extended Data Fig. 4a,b), and that all MNs share common synaptic input (Extended Data Fig. 4c,d) as previously proposed^18^. Tonic common excitatory synaptic drive to MN1–5, as induced by optogenetic stimulation, is therefore transformed into out-of-phase MN1–5 firing. Second, unpatterned, tonic, optogenetic stimulation of the five MNs (see Extended Data Fig. 5a,b for selective expression of transgenes in MN1–5) during flight increases their firing frequency and, therefore, wingbeat power, but it does not change phase relationships between MNs, as exemplified by the same characteristic gap around phase 0 for the MN4–MN5 pair (Extended Data Fig. 5). The strength of this dip around phase 0 in MN phase histograms is strongly correlated with full network splayness (definitions are provided in the Methods) in both simulated and experimental data (Extended Data Fig. 6). Similar phase histograms of MN pairs before and during optogenetic stimulation therefore indicate unaltered splayness. The generation of continuous splayed-out firing after selective, unpatterned, optogenetic stimulation of all five MNs indicates that interactions between MNs shape the firing patterns. An alternative possibility would be synaptic output from MN1–5 to feedback interneurons, which then generate the pattern. However, neither trans-synaptic mapping nor genetic markers for central output synapses from MN1–5 provide evidence for postsynaptic partners in the ventral nerve cord (VNC; Extended Data Fig. 7). Thus, it seems probable that the timing and pattern of MN activation does not require patterned activity of interneurons and, instead, the MN1–5 ensemble constitutes a minimal CPG.

One possible mechanism to transform common excitatory input to an ensemble of MNs into dispersed firing is lateral inhibition among MN1–5 by chemical synapses^15^. We reject this possibility by combining targeted genetic manipulation of MNs with in vivo recordings during flight. Knockdown (KD) of receptors for inhibitory transmitter (GABA A receptors (GABA-ARs) and glutamate-gated chloride channel (GluCl)) increases MN firing frequencies, but the phase relationships remain similar (Extended Data Fig. 6e,f). Lateral inhibition through chemical synapses is therefore not required for pattern generation.

**Fig. 2:**
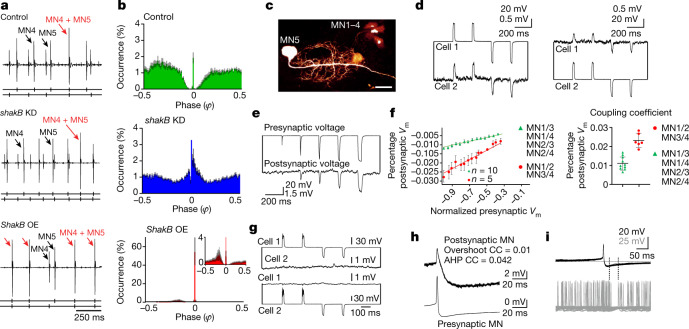
Electrical synapses shape CPG output by desynchronizing MN firing. **a**, Representative recording of MN4 and MN5 during tethered flight in the control (top) and after RNAi KD of *shakB-*encoded electrical synapses (DLM-Split-GAL4>UAS-shakB-RNAi, middle) and overexpression of ShakB in MN1–5 (bottom). The black arrows mark MN4 and MN5 spikes, and the red arrows indicate simultaneous MN4–MN5 spikes. **b**, Phase histograms of the occurrence of MN5 spikes (*y* axis) in relation to consecutive MN4 spikes (phase *φ* = 0 corresponds to the MN4 spike) for control (top), *shakB* KD (middle) and ShakB overexpression (bottom) with a magnified view (inset) (*n* = 10 animals for each genotype). Data are mean (coloured bars) ± s.e.m. (grey). **c**, MN1–5 dye coupling in the *dfmr1* RNAi KD background to increase dye uptake^28^. Scale bar, 20 μm. **d**–**h**, Intracellular recordings of MN pairs. **d**, Hyperpolarizations and depolarizations were conducted bidirectionally (from cell 1 to cell 2 and vice versa). **e**, Increasing current injection amplitude (top trace) increases response amplitudes in electrically coupled MNs (bottom trace). **f**, Plotting the mean presynaptic voltage (V_m_) area against the mean postsynaptic voltage area (left) reveals linear relationships, but regression slopes differ between distinct MN pairs (5 animals with strong coupling between the MN1–MN2 and MN3–MN4 pairs; 10 animals with weak coupling between the MN1–MN3, MN1–MN4, MN2–MN3 and MN2–MN4 pairs). The mean CC (postsynaptic peak voltage divided by presynaptic peak voltage; right) differs significantly between MN1–MN2, MN3–MN4 (red, 6 pairs) and all other pairs (green, 10 pairs). Data are mean ± s.e.m. (left) and mean ± s.d. (right). **g**, RNAi KD of *shakB* eliminates detectable electrical coupling between MNs (3 animals). **h**, The CC for the spike AHP is higher than for the spike overshoot. **i**, Firing of a coupled MN ceases during the AHP of the presynaptic MN.

Another possibility to create a neural network exclusively from MN1–5 is to connect them with electrical synapses. This has previously been suggested^22,23,24^, but experimental evidence has been lacking, and electrical coupling seems difficult to reconcile with desynchronized firing. We have tested this by genetic manipulation of innexins^25^, the invertebrate counterparts of connexins^26^, which comprise the pore-forming proteins of electrical synapses. ShakingB (ShakB) is the innexin expressed in the *Drosophila* escape circuit, including in MN1–5. KD of *shakB* using RNA interference (RNAi) in MN1–5 reduces electrical coupling below the detection threshold in paired patch-clamp recordings (Fig. 2g).

**Extended Data Fig. 8:**
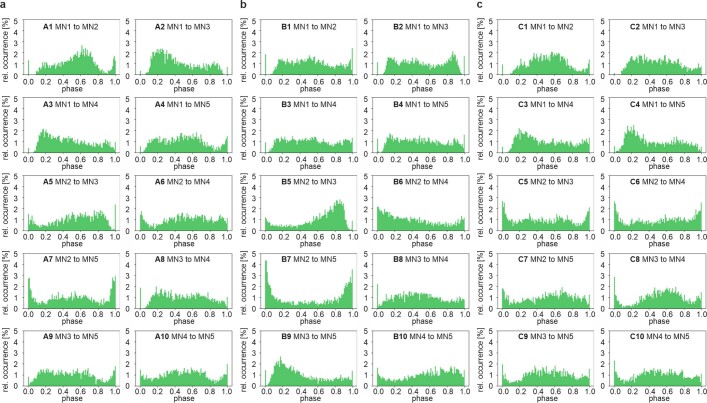
MN1–5 firing phase relationships require gap junctions (GJs) between MNs. For three individual male *Drosophila melanogaster* flies with targeted expression of RNAi knockdown for ShakB in MN1–5 **(a–c)** all 10 possible pairwise combinations of the MN1–5 are plotted as cumulative phase histograms from simultaneous recordings of MN1–5 during 10 min of continuous tethered flight (equalling approximately 3000 spikes of each MN). Starting with the MN1-MN2 pair on the upper left, **(a1-c1)**, for each MN pair, the interspike intervals between two consecutive MN spikes was normalized and divided into 100 equally sized bins (x-axis). Next it was determined in which bin the spike of the other MN occurred. This was repeated as sliding window for all interspike intervals and bins were filled cumulatively and normalized to total spike count (y-axis). In all three individuals with knockdown for ShakB mediated GJs in MN1–5 **(a–c)** the phase relationships that are characteristic for each MN pair in control flies (see Extended Data Fig. 1) and conserved across dipteran species (see Extended Data Fig. 2) are impaired.

Genetic manipulation of *shakB* in the five MNs disrupts the splay state without affecting the firing frequencies (Fig. 2a,b). As exemplified for the MN4–MN5 pair, in control animals (*n* = 7), firing of MN5 is inhibited before and after the occurrence of MN4 spikes (Figs. 2a,b (top)), therefore resulting in a characteristic gap around phase 0 (Extended Data Figs. 3b and 5e) that is indicative for network splayness (Extended Data Fig. 6a–d). By contrast, with *shakB* KD in MN1–5, the gap around phase 0 is absent and firing is more random with a slight preference for in-phase firing (Fig. 2a,b (middle)). Thus, electrical synapses are required for firing desynchronization in this small CPG. Recordings of all five MNs during flight confirm that *shakB* KD in MNs impairs their normal phase relationships and, therefore, the splay state (compare Extended Data Fig. 8 with Extended Data Fig. 1). This contradicts the common notion that electrical synapses function to synchronize network activity^27^.

Key to this role of gap junctions is weak electrical coupling, because increasing electrical coupling by overexpression of ShakB in MN1–5 causes firing synchronization (Fig. 2a,b (bottom)). Thus, the in vivo data indicate that weak electrical synapses cause firing desynchronization, but stronger coupling synchronizes firing. Weak electrical coupling between MN1–5 is also supported by anatomical experiments. Although we have previously not observed diffusion of small dye tracer molecules through gap junctions between MN1–5^16,21^, in *Drosophila* neurons, dye uptake during iontophoresis and subsequent diffusion through *shakB*-encoded electrical synapses are increased by KD of the fragile-X mental retardation protein (FMRP) (encoded by *dfmr1*)^28^. In this genetic background dye coupling of MNs is reliably observed (Fig. 2c).

In summary, a minimal CPG of five electrically coupled MNs is sufficient to pattern splayed-out firing across power demands and does not rely on additional interneurons or chemical synapses. First, lateral inhibition by chemical synapses is not required (Extended Data Fig. 6e,f); second, we found no evidence for chemical output synapses from MN1–5 in the VNC (Extended Data Fig. 7); third, unpatterned optogenetic activation of either presynaptic cholinergic neurons or the MNs during tethered flight increases MN1–5 firing rates and wingbeat frequency (Extended Data Figs. 3 and 5), but the phase relationships between MNs remain unaltered (compare Fig. 2a with Extended Data Figs. 3b and 5e). Finally, weak electrical synapses between MNs are required to generate normal phase relationships (Fig. 2a,b). However, this raises the question of how electrical synapses cause firing desynchronization.

## Electrical coupling within the CPG

A first step in deciphering the mechanism is to characterize the electrical synapses. Dual in situ patch-clamp recordings of MN pairs confirm electrical coupling and demonstrate that the electrical synapses are weak, bidirectional and non-rectifying (Fig. 2d). Non-rectifying, because depolarizing and hyperpolarizing current injections (20 ms duration) into one MN cause gap junctional potentials in the other MN. The relationship between presynaptic and postsynaptic charge is linear (Fig. 2e,f). Bidirectional, because the direction of charge transfer can be reversed (Fig. 2d). Compared with common CCs (postsynaptic charge/presynaptic charge^29^) estimated in different types of neurons^29^ (0.02–0.2), electrical synapses between the five MNs are weak, but coupling is twice as strong for the MN1–MN2 and MN3–MN4 pairs (coupling coefficients (CC) = 0.023 ± 0.003) compared with all of the other possible combinations of MN1–4 pairs (CC = 0.01 ± 0.0027; Fig. 2f).

The possibility that electrical synapses may desynchronize neural networks under specific conditions has been suggested by theoretical work^30–33^. Experimentally, a transient desynchronization of electrically coupled cerebellar Golgi cells has been described in one study, but for the specific condition of sparse input^34^. There, the transient desynchronization is attributed to a more effective gap junctional transmission of the afterhyperpolarization (AHP) of the action potential compared with its depolarizing overshoot^34^. Similarly, we found that the AHP of MN spikes is transmitted more effectively (CC, 0.042 ± 0.04) through ShakB-mediated electrical synapses than the brief spike overshoot (CC, 0.01 ± 0.004; Fig. 2h). CCs can differ for different components of the action potential because the duration of the presynaptic signal and the time constant of the postsynaptic membrane shape the junction potential^29^. Paired in situ current-clamp recordings of MNs that were induced to fire tonically by somatic current injection indicate that firing of one MN can depress firing of the other during and shortly after the AHP (Fig. 2i). However, the preconditions for desynchronization, as described for electrically coupled cerebellar Golgi cells^34^, are probably not fulfilled in the insect asynchronous flight CPG. First, network desynchronization by electrical synapses during flight is not transient but permanent. Second, network desynchronization manifests not only under sparse synaptic input regimes, but through the full range of synaptic input that occurs during flight. Thus, although we cannot rule out a contribution of the AHP, a general mechanism for small network desynchronization without a pronounced AHP (which is probably not sufficient; see above) or inhibitory synapses (not necessary for MN desynchronization; Extended Data Fig. 6e,f) is required. Indeed, theoretical considerations of network connectivity as well as cell-intrinsic excitability provide insights into how the observed splay states are generated.

## Coupling strength and excitability class

According to the theory of coupled phase oscillators, splayed-out network states are obtained if pairs of neurons have a preference to fire out of phase, that is, they are phase-repulsive^24^. However, for networks of more than two neurons, a strict antiphase state that maximizes the difference between the firing phases of two neurons cannot be achieved across all neuronal pairs; these systems are therefore called frustrated^35,36^. Typically, they can organize into a splay state that equidistantly maximizes phase distances among neurons to minimize frustration. The phase difference between individual pairs of neurons is therefore not maximal (that is, smaller than half the ISI), but firing phases of neuron pairs are still separated across the population of cells, which reflects the splay state that we observe in vivo (Fig. 1a,f,g and Extended Data Figs. 1 and 6a–d).

**Fig. 3:**
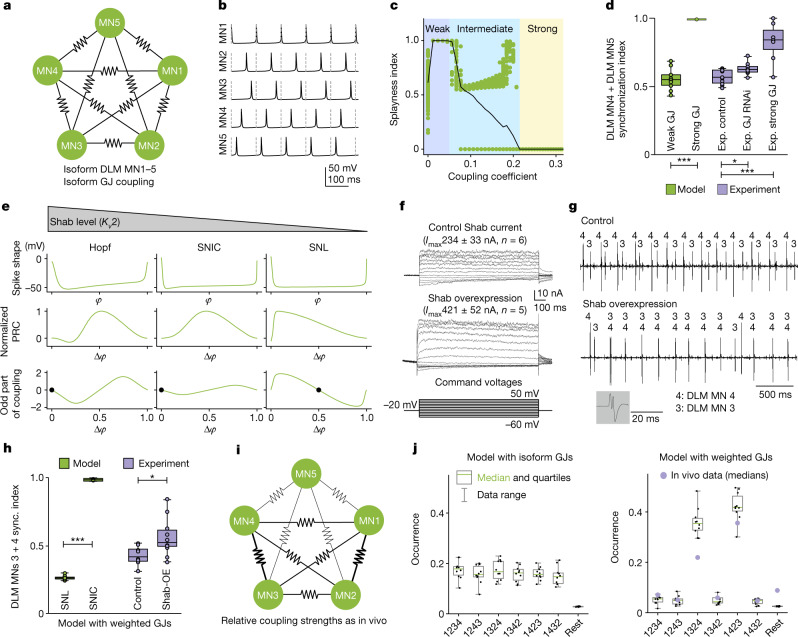
Weak electrical synapses and saddle homoclinic orbit (HOM) excitability dynamics underlie firing desynchronization. **a**,**b**, Conductance-based model with identical gap junctions (GJs, coupling coefficient CC < 0.05) between MN1–5 (**a**) produces splayed-out firing (**b**). **c**, Weak CCs (<0.05) cause high splayness indices (Methods); intermediate CCs (0.05–0.21) decrease splayness and yield multiple stable network states; CCs of >0.21 yield network synchrony (splayness = 0; 200 simulations per CC, green dots; the black line shows the average). **d**, Quantification of 60 s simulations (green, 10 simulations per condition) with a weak CC (0.005) yields significantly lower MN4–MN5 synchronization indices (Methods; median = 0.54) compared with strong coupling (CC = 0.258; median = 1.0; two-sided Mann–Whitney *U*-test, *P* = 0.0002). Similarly, experimental (exp.; purple) synchronization indices are significantly lower in the controls (median = 0.56, 11 animals) compared with the ShakB-overexpression group (median = 0.84, 7 animals, *P* = 0.0008). **e**, Increasing Shab channel levels transforms the single-MN model dynamics from HOM near the SNL point through SNIC to Hopf types (Extended Data Fig. 9a), which vary in action potential waveform (top) and PRC (middle). Averaging theory (Methods and equation (4)) yields the odd part of the coupling function (bottom) for each phase distance ∆*φ*, of which the fixpoints (black dots) determine stable network states. Only the SNL type yields one stable fixpoint at phase 0.5, therefore favouring anti-phase firing. **f**–**h**, Shab overexpression in MN1–5 nearly doubles Shab current (**f**), which causes in-phase firing of the MN3–MN4 pair (**g**) and significantly (two-sided Mann–Whitney *U*-test, *P* = 0.0434) increased synchronization indices (median = 0.52, 10 animals) compared with the controls (median = 0.43, 8 animals) (**h**). Similarly, increasing Shab in models with weighted GJs (as in Fig. 3i) significantly increased MN3–MN4 synchronization (sync.) indices (median = 0.99, *P* = 0.0002). **i**,**j**, Network models with heterogenous CCs (**i**) as found in vivo (Fig. 2f) yield preferred splay states as in animals (**j**, right; 10 simulations per condition; 8 animals, the purple dots depict median values of in vivo data), whereas homogenous coupling does not (**j**, left). For the box plots in **d**, **h** and **j**, the median (centre line), quartiles (box limits) and range (whiskers) are shown.

**Extended Data Fig. 9:**
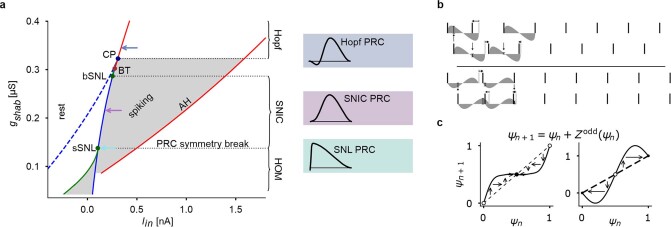
Motoneuron model analysis and phase-reduced view on coupling. **(a)** Codim-2 bifurcation diagram of the motoneuron model with focus on global bifurcations that switch the PRC shape with changing Shab levels (y-axis, *g*_shab_). Small and big saddle-node loop (sSNS, bSNL) encapsulate the SNIC interval, where spiking (grey area) commences after a saddle-node bifurcation (blue line) on a limit cycle. Saddle Homoclinic loop (HOM) line is green. The Andronov-Hopf bifurcation (AH, red line), ends in a Bogdanov-Takens (BT) point. At the cusp (CP) the neuron model switches from 3 to 1 fixpoints. The three PRCs (insets, right) show distinct symmetry properties. **(b)** Simplified illustration how asymmetric PRC components induce stable phase relations. With spike shapes in the form of delta functions, the odd parts of the PRC and of the coupling function have the same shape. The mean PRC component is absorbed into the average firing rate and plotted is only the PRC’s odd part (grey). Upon perturbations (arrows) PRCs with phase-advance in the ISI’s first half and phase-delay in the second half cause anti-phase synchronization (top), while the reverse causes in-phase synchronization (bottom). **(c)** Iterated maps (cobweb plots) illustrate the qualitative behaviour of phase differences. Starting at a random phase difference, *ψ*_*n*_, a vertical arrow is drawn from the diagonal to the function curve, constituting the new phase, *ψ*_*n*+1_, after a perturbation. From this point a horizontal arrow is drawn back to the diagonal, such that *ψ*_*n*_ is substituted by *ψ*_*n*+1_. Repeating these two steps, the system converges to stable fixpoints in phase differences (according to equation (3); Methods). In the case of a PRC with phase-advance in the ISI’s first half and phase-delay in the second half (left), the fix point is *ψ* = 0.5, while for the reverse case (right) the fix point is *ψ* = 0.

Whether pairs of neurons tend to fire out of phase depends on the specific combination of synaptic connectivity and cellular excitability (in other words, the dynamics of action potential generation). Cellular excitability classes characterize qualitatively different types of spiking dynamics and are associated with distinct mathematical bifurcation types at spike onset (that is, threshold). Given the weak electrical coupling among MN1–5, in theory, repulsive phase coupling^24,33,37,38^ can be fostered by specific excitability classes. To examine this hypothesis, we use a three-dimensional conductance-based neuron model fitted to MN sodium and delayed rectifier ion channel kinetics^39^ (Methods) to generate a gap junctional network of MN1–5 (Fig. 3a). Subjecting the single neuron model to a mathematical bifurcation analysis within the physiological parameter range identified a cellular excitability class that our analysis predicts to favour out-of-phase firing (through a homoclinic spike-onset near the saddle-node loop (SNL) bifurcation; Methods and Extended Data Fig. 9a). Indeed, five identically, weakly electrically coupled MNs with cellular dynamics near the SNL point robustly exhibit a desynchronized splay state (Fig. 3b). Note that the minimal conductance-based model does not contain a pronounced AHP (Fig. 3b (spike shape)), showing that the presynaptic AHP is not mandatory for network desynchronization.

By contrast, systematic variation in electrical coupling strength reveals that weak electrical coupling is required because models with non-zero, small CCs (<0.05), as obtained in vivo (Fig. 2f), yield splayed-out firing (Fig. 3c), whereas, for CCs between 0.05 and 0.21, network in-phase synchronization increases and, for CCs of >0.21, the network state is synchronized (Fig. 3c). To test these model predictions experimentally, we manipulated gap-junction strength genetically and quantified the synchrony of the MN4–MN5 pair from in vivo recordings during flight (Fig. 3d). In control animals with weak gap junctions, the synchronization index (Methods) is low, similar to model simulations with weak gap junctions. RNAi KD of electrical synapses increases synchronization in vivo (Fig. 3d), underscoring that gap junctions are required for desynchronization. Finally, the model predicts synchrony for strong electrical coupling. Indeed, strengthening of electrical coupling by ShakB overexpression significantly increases synchrony in vivo (Fig. 3d; see also Fig. 2b). Thus, weak electrical coupling is required for network desynchronization.

## The mechanistic core of the splay state

We next examined why cellular excitability dynamics close to the SNL point favour splay states. For regularly firing cells with all-or-none action potentials, three main dynamical excitability classes—shaped by cell-intrinsic properties including the ion channel composition—exist^40^. Mathematically, these correspond to three distinct spike-onset bifurcations: the subcritical Hopf, the saddle-node-on-an-invariant cycle (SNIC) and the saddle homoclinic orbit (HOM) bifurcations (Fig. 3e (top row)), which in turn qualitatively determine the phase dependence of a neuron’s sensitivity to inputs (also termed the phase-response curve (PRC)) in a bifurcation-specific and, therefore, excitability-class-specific manner (Fig. 3e (middle row)). For the network state, the coupling function, which combines the influence of the PRC and electrical coupling, is decisive (definitions are provided in the Methods), therefore assigning a direct impact on network dynamics to both gap junctions and cellular voltage dynamics^38^. Specifically, an asymmetry of the PRC shape favours stable phase relationships of spiking in the network; these are predicted by the fixpoints of the coupling function’s odd part (that is, fixpoints that can be read off the asymmetric components of the function; Methods). Our analysis shows that, for weak reciprocal electrical coupling, out-of-phase firing of identical neurons can be obtained only in combination with a PRC that is monotonically decreasing around phase 0.5 (a graphical explanation is provided in Extended Data Fig. 9b,c). Such a PRC is found for HOM excitability, including dynamics close to the border of SNIC firing, the latter corresponding to the SNL point introduced above (Fig. 3e (middle row)). Here, weak electrical coupling combined with such cellular dynamics results in a stable fixpoint at out-of-phase firing (Fig. 3e (bottom row)). By contrast, for the other two spike-onset bifurcations, synchronous in-phase firing is expected (compare also the stable fixpoints for the phases between neurons; Fig. 3e (bottom row)). Precisely, we predict that MN1–5 should dynamically reside close to the SNL point. Here, the PRC shape enables a network splay state, yet the slope of the neurons’ firing-rate versus current-input (*f*–*I*) curves is not as steep as deeper in the HOM regime, therefore enabling smooth control of MN firing frequency and wingbeat power, as observed in vivo (Fig. 1c–e).

**Extended Data Fig. 10:**
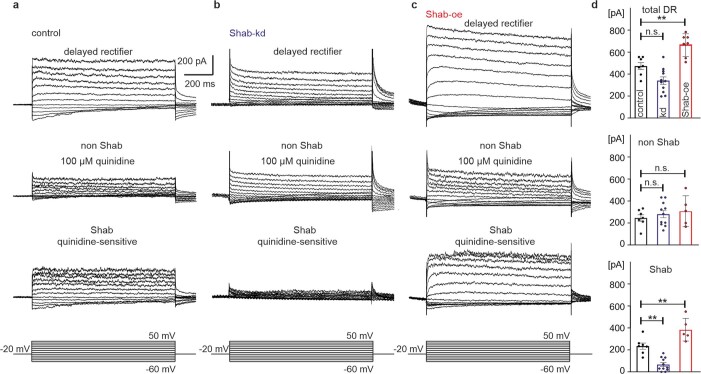
Shab constitutes 50% of the delayed rectifier current in MN1–5. **(a)** Outward current as measured in control MNs in TTX containing (10^−7^ M) calcium free saline by applying command voltage steps from a holding potential of −20 mV down to −60 mV and up to +50 mV in 10 mV increments (top traces). Same recording in 100 µM quinidine (middle). Bottom traces show the quinidine-sensitive Shab current by subtraction of the top minus the middle traces. **(b)** Same experiment but following Shab RNAi-kd (Shab-kd) in MN1–5. **(c)** Same experiment but following UAS-Shab overexpression (Shab-oe) in MN1–5. **(d)** Quantification of total delayed rectifier (DR) outward current (top), quinidine insensitive, non-Shab DR current (middle), and Shab DR current (bottom) in control (black, 6 replicate animals), with Shab-RNAi (blue, 11 replicate animals), and following Shab-overexpression in MN1–5 (red, 7 replicate animals, 5 for isolated Shab and non Shab current). In control, Shab constitutes ~50% of the DR current. Shab-RNAi significantly reduces Shab current by ~70% (ANOVA with Tukey’s posthoc comparison, p = 0.0016), but does not affect quinidine insensitive non Shab current (ANOVA with Tukey’s posthoc comparison, p = 0.8215) or total DR current (ANOVA with Tukey’s posthoc comparison, p = 0.0608). Shab overexpression significantly increases Shab current amplitude (ANOVA with Tukey’s posthoc comparison, p = 0.0029), significantly increases total DR current (ANOVA with Tukey’s posthoc comparison, p = 0.0010), but non Shab DR current remains unaffected (ANOVA with Tukey’s posthoc comparison, p = 0.6291).

To test this model prediction, the excitability class of MN1–5 has to be manipulated in vivo. Shab potassium channels constitute around 50% of the delayed rectifier current in these MNs (Extended Data Fig. 10). Bifurcation analysis of the single-cell MN model reveals an SNL bifurcation (Extended Data Fig. 9a). Moreover, increases in total Shab channel conductance, *g*_shab_, induce several transitions in excitability class^37^, from HOM (at low *g*_shab_), through the SNL point into a SNIC (medium *g*_shab_) and a Hopf bifurcation (high *g*_shab_, Fig. 3e and Extended Data Fig. 9a). At both excitability dynamics resulting from higher *g*_shab_ (SNIC and Hopf), the weakly coupled MN1–5 network exhibits a synchronized state, whereas SNL dynamics favour splay states (as described above). We tested this model prediction experimentally by genetic manipulation in vivo. Targeted overexpression of Shab in MN1–5 causes a near doubling of Shab delayed rectifier current (Fig. 3f) without compensatory changes in the amplitudes of non-Shab delayed rectifier current (Extended Data Fig. 10). Recordings of the MN3–MN4 pair were used to test whether this caused firing synchronization in vivo. In controls, both MNs fire out of phase (Fig. 3g (top) and Extended Data Fig. 1). By contrast, with Shab overexpression, the MN3–MN4 pair shows markedly increased synchronization (Fig. 3g (bottom)). Variability in synchronization strength between animals (Fig. 3h) is probably caused by different overexpression strengths and variable levels of other delayed rectifier channels. We also cannot exclude some compensatory regulation of yet other currents. However, pooling the data from all recordings (*n* = 10) revealed a significant increase in firing synchrony after overexpression of Shab (Fig. 3h), as predicted by an increase in *g*_shab_ in the network model (Fig. 3h). By contrast, our model predicts that a decrease in *g*_shab_ shifts MNs deeper into the HOM regime but does not cause a transition in excitability class (Fig. 3e and Extended Data Fig. 9a). Thus, with reduced *g*_shab_ in MN1–5, the network model predicts desynchronization (Fig. 3e). We tested this prediction experimentally. Compared with the control (*n* = 7 animals) targeted RNAi KD of *Shab* in MN1–5 (*n* = 11 animals) reduced the Shab current by around 70% (Extended Data Fig. 10). Paired in vivo recordings of MN4 and MN5 during flight confirm a low synchronization index as predicted by the model (median synchronization indices are 0.559 for control flies (*n* = 11 animals) and 0.598 for *Shab* KD (*n* = 11) and were not statistically different; *P* = 0.393, *U* = 74, two sided Mann–Whitney *U*-test).

The theoretical principles underlying this mechanism are independent of model details (instead they depend on the dynamical excitability class). Weak electrical coupling between neurons with SNL excitability therefore provides a generic mechanism that suffices to produce the observed desynchronized (splay) states in small networks. Importantly, this mechanism holds across different firing rates and does not require a pronounced AHP, but the AHP may further stabilize desynchronization. Although previous studies have provided mechanisms by which networks with electrically coupled neurons can be configured into desynchronized, phase-locked firing^41^—such as inhibitory synapses overriding the synchronization by electrical synapses^42^ or interacting with the spiking synchrony^43,44^, silencing of electrical synapses in a network with mixed chemical and electrical synapses^42,45^, or strongly asymmetric electrical synapses and specific input regimes^46^—desynchronization by the electrical synapse alone, without the need of additional chemical synapses, other network motives or feedback from the network, is conceptually new.

## Sequence preference requires heterogenous coupling

Our in vivo recordings show that MN1–5 firing is not only desynchronized, but organized into preferred sequences of cellular activation (within the splay state; Fig. 1f,g). This raises of the question of how the preferred sequences are generated. Our modelling (5 min simulations in network models with SNL neurons and noise) demonstrates that homogenous electrical coupling does not show sequence preference (Fig. 3j (left)). By contrast, heterogenous electrical coupling between MN1–5 (Fig. 3i) as observed in situ (Fig. 2f) produces the same preferred splay states with similar sequence statistics (Fig. 3j (right)) as observed in vivo across animals (Fig. 1f). We conclude that heterogenous weak electrical coupling among neurons with HOM excitability (near SNL) sufficiently explains the preferred splay states in this small network.

## Splay state serves stable wing power

**Fig. 4:**
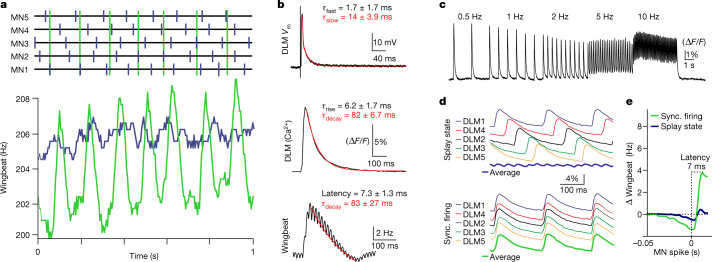
Splayed-out firing ensures stable wingbeat power. **a**, Representative traces of wingbeat and MN multiple-unit recordings during flight (7 animals in each condition). Synchronous MN firing (top, green) causes around eight times larger fluctuations in wingbeat frequency (bottom, green) compared with splayed-out firing (blue). **b**, DLM fibre voltage response to one MN spike is fast (top; decay *τ*_fast_ ~ 1.7 ms, *τ*_slow_ ~14 ms; 3 animals). Both the resulting myoplasmic calcium signal (middle; GCaMP8f ∆*F*/*F*; rise *τ* = 6.2 ± 1.7 ms; decay *τ* = 82 ± 6.7 ms; 7 animals) and wingbeat frequency changes after MN spiking (bottom; latency = 7.3 ± 1.3 ms; decay *τ* = 83 ± 27 ms; 7 animals) follow similar slower time courses. **c**, DLM6 calcium responses to different MN5 firing frequencies (Supplementary Video 4). **d**, As each DLM fibre is innervated by one MN, splayed-out firing must result in time-shifted calcium responses (top), but synchronous MN firing results in time-locked calcium responses across fibres (bottom). The resulting average myoplasmic calcium levels across all fibres is considerably larger for synchronous (bottom panel, bottom trace) compared with for splayed-out firing (top panel, bottom trace). **e**, As a result, during flight, splayed-out firing is accompanied by small transient fluctuations in wingbeat frequency (blue; *n* = 7 animals) but synchronous firing is accompanied by significantly (*P* = 0.0010, two-sided unpaired *t*-test) larger fluctuations with a latency of around 7 ms after each MN spike (green; *n* = 7 animals).

MN1–5 splayed-out firing in multiple species suggests that it provides a functional benefit. We tested this hypothesis by comparing the wingbeat frequency between normal splay state and synchronous MN firing, the latter induced by increasing electrical coupling through genetic manipulation (Figs. 2a and 3c). In vivo, synchronous MN firing causes 8× higher wingbeat frequency fluctuations over time compared with firing in splay state (Fig. 4a). The amplitudes of such fluctuations are close to changes in wingbeat frequency in response to optomotor input^1,16^ and are therefore functionally relevant for flight altitude control. In the steady-state, muscular stretch sensitivity and wingbeat frequency are directly proportional to the myoplasmic calcium levels^1^, but the dynamics of myoplasmic calcium in DLM fibres is unclear. To link MN firing patterns to wingbeat frequency, we measured the kinetics of muscle fibre membrane potential and myoplasmic calcium concentration changes after MN1–5 firing (Fig. 4b). An MN spike causes a rapid depolarization of the DLM fibre membrane (Fig. 4b (top)), which is followed by a myoplasmic calcium signal with a rise time constant of around 6 ms and a decay time constant of about 80 ms (Fig. 4b (middle)). If muscular stretch sensitivity was controlled by the myoplasmic calcium levels with a ms temporal resolution, changes in wingbeat frequency during simultaneous MN firing should follow the same time course as changes in myoplasmic calcium across all six DLM fibres. This is precisely what we found (Fig. 4b (bottom)). Synchronous MN firing causes peak wingbeat frequencies after 7.3 ± 1.3 ms (myoplasmic calcium rise time constant *τ* = 6.2 ± 1.7 ms) that decline with a time constant *τ* = 83 ± 27 ms (calcium decay time constant is 82 ± 6.7 ms).

To determine the kinetics of myoplasmic calcium also at different MN firing frequencies, we stimulated a single MN electrically at different frequencies (0.5, 1, 2, 5, 10 Hz), spanning the normal range during flight, and imaged the resulting calcium signals in the respective DLM target fibre. Discrete myoplasmic calcium signals were observed for the entire frequency range tested (Fig. 4c and Supplementary Video 4), although summation as caused by an incomplete return to resting calcium levels between MN spikes starts at 2 Hz and increases at higher frequencies. However, for firing frequency ranges of MN1–5 that are observed during normal flight (Fig. 1c), in each muscle fibre, myoplasmic calcium fluctuates around each corresponding MN spike. Given that we did not observe any differences in the temporal coupling of MN firing and myoplasmic calcium across different stimulation frequencies or different DLM fibres, these data can be extrapolated to all DLM fibres for splay state (Fig. 4d (top)) as compared to synchronous MN firing (Fig. 4d (bottom)). Given that each DLM fibre is innervated by one MN only (Fig. 1a), MN firing in splay state causes splayed-out calcium signals across the different muscle fibres (Fig. 4d (top)), each peaking around 6 ms after the respective MN spike and declining with a time constant of around 80 ms (Fig. 4b (middle)). The bottom trace shows the resulting average myoplasmic calcium across fibres (Fig. 4d (top)). By contrast, during synchronous firing of MN1–5, the calcium signals in all DLM fibres are time locked, which results in much larger calcium fluctuations across DLM fibres (Fig. 4d (bottom)). This extrapolation of the average myoplasmic calcium levels across the DLM fibres predicts eightfold smaller fluctuations in wingbeat frequency over time in normal splay state than for synchronous MN firing. We confirmed this prediction by averaging changes in wingbeat frequency around MN spikes for splayed-out (Fig. 4e (blue line)) as opposed to synchronous firing (Fig. 4e (green line)). Thus, the splay state serves to minimize wing power fluctuations.

Taken together, splay-state asynchronous flight motor patterns are conserved across individuals and species, are produced by a minimal CPG of weakly electrically coupled MNs, and serve constant wingbeat power output at a given power demand. This provides a comprehensive view of the asynchronous flight CPG network structure and the resulting functional consequences for one of the most abundant forms of locomotion on earth. Moreover, we provide a theoretical background for firing d esynchronization/synchronization in small gap-junctional networks in the context of their specific cellular excitability. The underlying mechanism is generic, predicting desynchronizing functions of electrical synapses beyond the motor system of insects. Electrical synapses can therefore be used for operations such as sign-reversal and ensemble firing desynchronization, a functional versatility and impact on neural circuit dynamics that was previously attributed to chemical synapses alone.

## Methods

### Animals

*D. melanogaster* were reared in 68 ml transparent food vials (Kisker Biotech, 25 mm × 95 mm) filled with 10 ml of standard cornmeal/glucose/yeast/agar diet at 25 °C and 60% humidity under a 12 h–12 h light–dark cycle. For experiments, adult 2–5-day-old male flies were used. We used Canton-Special (Canton-S) as the wild-type control. To selectively target the DLM MNs (MN1–5) with UAS-driven transgenes we used a split GAL4 driver line that combines GMR23H06 (BDSC, 49050 discontinued) and GMR30A07 (BDSC, 49512) from the Rubin Collection^47^. The GMR23H06 enhancer expresses the activating domain (AD) and the enhancer GMR30A07 expresses the DNA-binding domain (DBD). Overlap of both expression patterns—and, therefore, functional GAL4 expression—is restricted to the MN1–5 (Extended Data Fig. 5a,b). The following transgenes were expressed in MN1–5 to manipulate gap junction expression and function. *shakB* RNAi (BDSC, 57706) targets all *shakB* isoforms and was used to KD gap junctions in MN1–5. For gain of function through overexpression of gap junction protein the shakB(N+16) isoform was used (gift from P. Phelan). KD of inhibitory synaptic transmission through GluCls or RDL GABA-ARs was conducted by targeting the expression of UAS-RNAi^KK^ for GluClα (VDRC, 105754) or UAS-Rdl-RNAi (VDRC, 41103) to MN1–5 with the DLM MN GAL4 driver GMR23H06. To test for electrical synapse function by genetic manipulation, UAS-RNAi-KD of *shakB* was compared with UAS-ShakB overexpression and empty UAS-control with 7 biological replicates in each group. To test for effects of GABA-ARs and GluClRs on flight patterns, UAS-RNAi-kd for each receptor type was expressed in MN1–5 and the effect on flight patterns was compared with the respective genetic controls, with 8 biological replicates for GluClRs and 10 replicate animals for GABA-ARs. For all genetic manipulations, the order of experiments was fully randomized and analysis was conducted without knowledge of the genotype. Other insect species were obtained from pet shops as feed insects and *Apis mellifera* (at 4 to 6 weeks of age) from the local apiculture of the Johannes Gutenberg University Mainz.

For in vivo optogenetic manipulation of the activity of MN1–5 during flight, we expressed UAS-XXL channelrhodopsin (XXL-ChR) (BDSC, 58374)^48^ either under the control of our DLM-MN-specific split GAL4 driver (split GMR23H06+GMR30A07) to enable light activation of MN1–5 (7 replicate animals) or, alternatively, in presynaptic cholinergic neurons under the control of Cha-GAL4 (11 replicate animals). Direct light activation of MN1–5 during flight was used to test whether the CPG correctly shapes MN1–5 firing into preferred sequences even when firing rates are artificially increased. Light activation of presynaptic cholinergic neurons during flight was used to test whether the CPG translates artificially presented, unpatterned synaptic input into patterned output from MN1–5. For in vivo calcium imaging in DLM fibres (6 replicate animals) the genetically encoded calcium indicator UAS-GCaMP8f^49^ was expressed under the control of Act88-GAL4^50^.

We used targeted expression of *FMRP*^*RNAi*^ in MN1–5 (*w;UAS-FMRP*^*RNAi*^*/23H06-ADZ* *UAS-CD4-td-GFP;30A07-DBD/+*) to visualize weak electrical coupling by dye coupling. A reduction in FMRP is reported to increase both the uptake of small tracers into *Drosophila* neurons during iontophoresis and, consequently, also increase dye uptake into neurons that are electrically coupled through *shakB*-encoded gap junctions^28^.

No ethical oversight was required for work with insect species. The work was according to the guidelines of research with transgenic invertebrate animals of the state of Rhineland-Palatinate in Germany.

### Generation of UAS-Shab flies

*Shab* cDNA was provided by F. Sigworth^51^ in pBluescript KSM. *Shab* was then PCR amplified with the following primers:

TTCAGGCGGCCGCGGCTCGAGaacttaaaaaaaaaaatACAA**ATG**GTCGGGCAATTGCAAGG (forward primer; the XhoI restriction site is underlined, the *Shab* start is bold and underlined) and CCTTCACAAA**GAT**CCTCTAGAgactcactatagggcgaattgg (reverse primer; the XbaI restriction site is underlined, the *Shab* terminal sequence is bold and underlined). The primers included overhangs matching the pJFRC81 attB-site-directed expression vector (36432, Addgene). pJFRC81 was cut with XhoI and XbaI restriction enzymes according to the provider’s protocol (New England Biolabs, R0146S, R145S) to remove the GFP sequence from the vector. Using NEBuilder according to provider’s instructions (New England Biolabs, E5520S), the amplified *Shab* cDNA was inserted into the cut pJFRC81 vector, thereby replacing the GFP. The PCR-amplified *Shab* cDNA was not purified before assembly with NEBuilder. The assembled construct was then transformed into *Escherichia coli* Dh5α bacteria and streaked out onto ampicillin (50 µg ml^−1^) containing LB media agar plates. Overnight colonies were picked and grown in ampicillin containing LB media (50 µg ml^−1^). DNA was isolated using the Maxiprep kit (Qiagen, 12162). Using specific primers, we determined that the cDNA used was *Shab* splice variant K (Supplementary Table 5). The correct sequence was confirmed by sequencing. For generation of transgenic flies, the Shab-pJFRC81 attB vector was injected into fly embryos at the attP2 landing site by BestGene. For experiments, homozygous male w;;10xUAS-Shab- pJFRC81attP2 flies were crossed to female w;GMR23H06-ADZ;GMR30A07-DBD (DLM-split GAL4 driver) flies and male F_1_ progeny were tested.

### Electromyography/extracellular recording of MNs

The in vivo activity of MN1–5 during tethered flight was monitored by extracellular voltage recordings from their respective muscle fibre. One MN action potential causes one large, transient postsynaptic depolarization (Fig. 4b (top trace)) in its target DLM fibre (for innervation, see Fig. 1a). As each DLM fibre is innervated by one MN only, and each MN spike causes the same distinct evoked postsynaptic response, the EMG spikes recorded from the DLM fibres are a 1:1 reflection of the activity patterns of MN1–5.

To prepare flies for in vivo EMG recordings during tethered flight, animals were briefly cold-anaesthetized (20 s in an empty 68 ml plastic vial on ice), transferred dorsal side up onto a cold metal plate (~3 °C) and glued (clear glass adhesive (Duro; Pacer Technology)) with head and thorax to a triangle-shaped tungsten hook (0.1 mm diameter). Curing of the glass adhesive was induced by exposure to ultraviolet light (Mega Physik Dental Cromalux-E Halogen Curing Light Unit) for 45 s. The flies were kept individually for 10 min to recover from the cold anaesthesia. The rested flies were then mounted in the set-up to a clamp attached to a micromanipulator. Tarsal contact to a small polystyrene bead prevented unwanted engagement into flight. Next, a light barrier, consisting of a red laser, an aperture to reduce beam width to approximately 1 mm and a LED light sensor, was positioned so that the light beam was broken by each wingbeat. After positioning of this laser-based wingbeat detector, tungsten electrodes for extracellular MN recordings were inserted. A reference electrode was inserted into the last abdominal segment. To record the activity of MN5 and MN4 from the dorsal-most DLM fibres, we inserted one sharpened tungsten wire into the dorsal thorax in front of the anterior dorsocentral bristle, making sure not to cross the midline of the thorax. This resulted in extracellular recordings of two units that could easily be distinguished by amplitude and shape. Confirmation of unit identity was achieved by adjusting the tungsten insertion depths and monitoring the resulting amplitude changes of the recorded units. As MN5 innervates the dorsal-most DLM fibres, it is the first unit to appear after shallow dorsal electrode insertion. Moving deeper lets a second unit appear that belongs to MN4, which innervates the fibre just beneath. This MN4 unit increases in amplitude with increased insertion depth in DLM4, while the amplitude of MN5 decreases. This procedure allowed unambiguous allocation of the two units to MN5 and MN4. To record up to 5 DLM units (MN1–5) simultaneously, additional tungsten electrodes were inserted. A second electrode was inserted four small bristles in front of the first electrode, which recorded MN4 and MN3. A third electrode was inserted anterior on the same line as electrodes 1 and 2 at the position where small bristles begin to appear on the thorax. Again, unit identification was conducted by altering electrode insertion depth at the beginning of each experiment. Starting from dorsal and slowly moving ventrally, the first unit to appear is MN5, the second one is MN4, the third one is MN3 and so on. Electrode depths were adjusted until the subsequent unit that appeared while moving deeper had approximately half of the amplitude of the first unit, so that both were easily distinguishable. In some recordings, it was also possible to pick up three clearly separable units simultaneously, but spike sorting was more time consuming.

All extracellular recordings were amplified at 1,000× using the AM-Systems 1700 extracellular amplifier. Electrophysiological and wingbeat recordings as well as a frame trigger signal from high-speed video (see below) were digitized with the Axon Digidata 1550B (Molecular Devices), acquired with AxoScope (v.10.7) and stored on a PC. Spike sorting was conducted offline with the spike sorting function in Spike2 (v.7.2). Data were further analysed using Spike2, and custom Python routines were created using Jupyter notebook to count pattern probabilities and create phase histograms. For additional functions, the Python libraries NumPy, pickle, SciPy, Matplotlib and seaborn were imported. All of the Python scripts are available on request. Additional data analysis and statistics were conducted using Microsoft Excel Professional Plus 2019 (v.2110) and GraphPad Prism (v.9.2.0).

All of the animals that were successfully recorded for at least 10 min of flight were included in the analysis of phase relationships. For spike processing, the DLM recordings (.abf format) were imported into Spike2 and sorted with the wavemark function, therefore creating templates for each unit. Template-matching spikes were visualized separately and synchronous spikes (the sum of the amplitude and waveform of two distinct MN units) were split into the two according units. The timepoints of the peaks of all marked spikes were then imported into single-event channels. The first and last 3 s of each flight bout were discarded to restrict the analyses to stable in-flight motor patterns. At the beginning of flight bouts, the MN spikes occur at higher frequency and with the highest irregularity, whereas, at the end of a flight bout, the firing frequency decreases. Phase histograms were created from the event channels. Each ISI of an event channel with the timepoints of the units of a given MN was compared to when the units of a second MN occur within the ISIs and counted into bins. All ISIs were normalized to 1 (phase 0 to phase 1), divided into 100 bins, the events of the second MN unit were counted into the respective bins and divided by the total number of events (relative occurrence; Extended Data Figs. 1, 2, 6 and 8). To create phase histograms with the phase 0 in the middle of the diagram (Fig. 2b and Extended Data Figs. 3b, 5e and 6e,f), the last 50 bins were copied in front of the first 50 bins and the *x* axis was changed to range from phase −0.5 to phase 0.5.

All insect species larger than *Drosophila* were cold anaesthetized for up to 20 min in the fridge at 5 °C and fixated in a 3D printed device to hold the insect in place. The .stl file is available on request. Instead of a rectangular tungsten hook, the wire is bent in a half circle and glued onto the outer circumference of the posterior dorsal region of the thorax to increase the adhesive surface.

### High-speed video

In addition to electrophysiological recordings of MN activity and laser-based wing detection, selected times of tethered flight were simultaneously recorded as high-speed video using the Photron FASTCAM Mini UX100 camera and Photron FASTCAM Viewer software (PFV, v.3.6.9.0) at 5,000 fps. As illumination, two infrared lights (Sygonix IR illuminator with 48 LEDs) were used. Videos of *Drosophila* (Supplementary Video 1), goldfly (Supplementary Video 2) and honey bee (Supplementary Video 3) during tethered flight are provided.

### Electrolytic sharpening of tungsten electrodes

The tips of tungsten rods (diameter of 100 µm for *Drosophila*, 125 µm for all larger insects) were sharpened electrolytically by repeated dipping in a NaNO_2_ KOH solution (10.3 M NaNO_2_ and 6.05 M KOH in double-distilled H_2_O). Tungsten rods of 1 cm length were crimped onto metal rods. For this, the wire was placed onto the rod and covered with a 0.5 mm ferrule. A crimping tool compressed it to a pluggable tungsten wire electrode. Now the electrode was placed into an alligator clip, connected to a stimulator (Grass SD9 square pulse stimulator) and repeatedly dipped into the sharpening solution, which was connected to the other pole of the stimulator. A monophasic current with 100 Hz, 40 V and 1 ms duration was applied. The tip was frequently moved in and out of the solution to form a thin tip. Finally, electrodes were rinsed with distilled water.

### Double patch-clamp recordings of MN pairs

After removing the legs and wings, 2–3-day-old adult *Drosophila* males were pinned into a Sylgard-coated lid of a 35 mm Petri dish and fixed ventral side up with minute pins in the head and through the tip of the abdomen. After submerging in normal saline (128 mM NaCl, 2 mM KCl, 1.8 mM CaCl_2_, 4 mM MgCl_2_, 5 mM HEPES and ~35.5 mM sucrose, the pH was adjusted to 7.24 with 1 N NaOH, and osmolality was 300 mOsm kg^−1^) the thoracical cuticle was removed with fine iris scissors to expose the VNC. The specimen was then rinsed thoroughly with saline. After mounting the preparation onto the stage of an upright Zeiss Axio Examiner epifluorescence microscope, the VNC was viewed with a ×40 water-immersion lens (Zeiss W Plan Apochromat ×40/1.0 NA, DIC VIS-R), the UAS-6xmCherry-expressing MN1–4 (genotype: *w;23H06-ADZ* *UAS-6xmCherry/+;30A07-DBD/+*) were viewed through a TRITC filter set. The ganglionic sheath and debris hampering access to MN1–4 somata were removed by repeated application of 1% protease type XIV (from *Streptomyces griseus*, Sigma-Aldrich, P5147) through a patch pipette with a manually broken tip that was then also used to remove loosened debris^52^. Before recording, the specimen was washed with saline for 5 min through a gravitation perfusion system at around 2 ml min^−1^, the bath volume was around 300 µl. Recordings were performed using patch pipettes (borosilicate glass capillaries; outer diameter, 1.5 mm; inner diameter, 1 mm, without filament, World Precision Instruments, PG52151-4) pulled with a PC-10 vertical puller (Narishige) filled with internal patch solution (140 mM K-gluconate, 2 mM Mg-ATP, 2 mM MgCl_2_, 11 mM EGTA, 10 mM HEPES. pH was adjusted to 7.24 with 1 N KOH, osmolality was adjusted to 300 mOsm kg^−1^ with glucose if necessary) that approached the two MNs from opposite sides. Patch pipette tip resistance was between 5 and 6 MΩ with these solutions. Double patch recordings were performed by connecting each patch pipette to a separate Axopatch 200B amplifier (Molecular Devices). Data were filtered at 5 kHz through a lowpass Bessel filter, digitized through an analogue/digital converter (Digidata, 1440), and signals were recorded using pClamp10.7 software (both Molecular Devices). Output gain was 10×. For double recordings, one MN was approached with a patch pipette. After giga seal formation, pipette capacitance artifacts were cancelled manually and whole-cell configuration was established at a holding potential of −70 mV. After setting whole-cell capacitance compensation, correction and prediction values as well as series resistance compensation using the respective dials of the amplifier (only used to judge on recording quality as all of these compensations are disabled in current clamp mode), the recording was disconnected from external influence by using the I=0 setting of the amplifier, making it possible to revert to bath mode without disturbing the already established recording of the first MN. Then, recording of the second MN was established identically. To monitor both recordings, separate channels of the digitizer and in the pClamp10.7 software were used. Quality parameters were as follows: giga seal, >5 GΩ; membrane potential of −70 mV was held with a holding current smaller than ±100 pA; series resistances of >15 MΩ were not accepted to ensure good control when applying current injections; only if these criteria were met for both MNs, the recordings were switched to current clamp mode. Resting membrane potential was around −60 mV without current injection. To determine coupling strength and rectification parameters of gap junctions between MNs, depolarizing and hyperpolarizing current was injected into one MN while the other was monitored simultaneously, and vice versa. Slow tonic firing was induced in one or both MNs at rates of between 3 and 8 Hz, as is observed during flight behaviour, by small somatic current injections while monitoring the respective other MN. For input–output relationships, firing was induced by 1,000 ms square pulse current injections up to 1 nA in 0.1 nA increments.

### Intracellular dye filling

Intracellular dye fills with neurobiotin were used to detect dye coupling between electrically coupled MNs. For intracellular dye fills, *FMRP*^*RNAi*^ was targeted to MN1–5 (*w;UAS-FMRP*^*RNAi*^*/23H06-ADZ* *UAS-CD4-td-GFP;30A07-DBD/+*), because reductions in FMRP have been shown to increase both neurobiotin uptake into *Drosophila* neurons during iontophoresis and, consequently, also increased dye uptake into neurons that are electrically coupled through *shakB-*encoded gap junctions^28^. After removing the legs and wings, 2–3-day-old adult male *Drosophila* were pinned in a Sylgard-coated lid of a 35 mm Petri dish and fixed dorsal side up with two minute pins, one through the head and one through the abdomen. After submerging the specimen in normal saline (128 mM NaCl, 2 mM KCl, 1.8 mM CaCl_2_, 4 mM MgCl_2_, 5 mM HEPES and ~35.5 mM sucrose, pH was adjusted to 7.24 with 1 N NaOH, and osmolality was 300 mOsm kg^−1^), it was opened along the dorsal midline up to the neck connectives with iris scissors. The cut DLM was then pinned to the sides with one minute pin each to expose the gut, inner organs and VNC. The gut, salivary glands as well as other inner organs were removed to fully expose the VNC. The specimen was then rinsed thoroughly with saline to remove excess debris. After mounting the preparation onto the stage of an upright Zeiss Axio Examiner epifluorescence microscope, the VNC was viewed using a ×40 water-immersion lens (Zeiss W Plan Apochromat 40x NA 1.0, DIC VIS-R), the UAS-CD4-td-GFP-expressing MN1–5 were viewed through a FITC filter set. The ganglionic sheath was removed focally using a broken patch pipette filled with 1% protease type XIV (from *Streptomyces griseus*, Sigma-Aldrich, P5147) to allow access to MN somata^52^. Dye fills were performed using sharp glass microelectrodes pulled from filamented borosilicate glass capillaries (Sutter BF100-50-10; tip resistance, ~40 MΩ) with a Sutter P-97 Flaming Brown microelectrode puller. The tip was filled with a 50/50 mixture of TRITC-Dextran 3000 lysin fixable (Invitrogen, 3308) and neurobiotin (Vector Labs, SP-1120) dissolved in 2 M potassium acetate, and the shaft was then filled with 2 M potassium acetate leaving an air bubble between the dye-filled tip and the shaft to avoid dilution of the dye. MNs were impaled by a short buzz (which makes the electrode tip vibrate for a set amount of time, here around 40 ms) with a remote buzz connected to an Axoclamp 2B intracellular amplifier (Molecular Device) that was also used for dye filling. MNs were filled iontophoretically by positive current injection (between 0.5 and 1 nA) in bridge mode until the MN was judged to be filled, after around 10 min. The microelectrode was then removed, and the specimen was fixed with 4% paraformaldehyde at room temperature for 50 min, without shaking. This was followed by three rinses with PBS and three washes for 20 min PBS and six washes for 20 min with 0.5% PBS-Triton X-100 (PBT, Triton X-100, Sigma-Aldrich, T8787), with shaking. The preparation was then incubated with Streptavidin coupled to Alexa 647 (Thermo Fisher Scientific, S-21374) in 0.3% PBT at room temperature in the dark for 2 h with shaking. Streptavidin Alexa 647 was then removed, the preparation was rinsed several times with PBS and then washed three times for 20 min with PBS at room temperature in the dark with shaking. This was followed by an ascending ethanol series: 50%, 70%, 90%, 100% for 10 min each. The preparation was then mounted in methylsalicylate, topped with a high-precision (170 ± 5 µm) cover slip and sealed with clear nail polish. The dye fill was then visualized using the Leica TCS SP8 confocal laser microscope with a helium–neon laser. Alexa 647 was excited at 633 nm and emission was detected between 650 and 680 nm with a photomultiplier tube. Images were taken with a ×40 oil objective (NA 1.3) with a 1.75 digital zoom at a resolution of 1,024 × 1,024 pixels and a *z*-step size of 1 µm.

### Optogenetics

For optogenetic stimulation, UAS-channelrhodopsin was either expressed under the control of a DLM-MN-specific Split-GAL4 line (GMR23H06-ADZ attP49; GMR30A07-DBD attP2; see Extended Data Fig. 5a,b for expression patterns specifically in MN1–5), or under the control of ChaT-GAL4 in cholinergic neurons. We tested multiple different optogenetic transgenes. UAS-CsChrimson for red light activation caused few MN spikes followed by silence, probably caused by a depolarization block, and was therefore not suitable for optogenetic stimulation during ongoing flight. By contrast, blue light stimulation of channelrhodopsin XXL reliably increased MN firing frequencies during flight, and therefore was the most effective transgene for optogenetic activation experiments during flight. As a control, the blue light stimulation was applied in an identical manner to flies without expression of UAS-channelrhodopsin XXL (DLM-MN split-GAL4 and ChaT-GAL4 crossed to w1118 flies, respectively). Blue light stimulation alone had no effect on MN firing frequencies during flight. The light stimulus was applied though fibre optics directed to the ventral thorax from underneath to avoid stimulation of cholinergic neurons in the brain. The light stimulus was presented constantly so that optogenetic activation of either cholinergic pre-motor neurons (Extended Data Fig. 3) or MN1–5 (Extended Data Fig. 5c,d) was not patterned. Moreover, channelrhodopsin XXL activates within milliseconds after blue light stimulation but has very slow inactivation kinetics with an off time constant of 76 ± 12 s (ref. ^48^). Thus, increased firing rates during flight outlast the blue light stimulus (Extended Data Figs. 3 and 5c).

### In vivo calcium imaging from DLM fibres during flight

For in vivo calcium imaging in DLM fibres, the genetically encoded calcium indicator UAS-GCaMP8f^49^ was expressed in muscle under the control of Act88-GAL4^50^. All earlier GCaMP versions exhibit signal decay kinetics that are too slow to measure the time course of decay of myoplasmic calcium signals as induced by a MN action potential. Calcium imaging was conducted using a Hamamatsu CMOS (C11440-42U) camera mounted to a Zeiss Axioscope 2FS fluorescence microscope equipped with a ×10 lens that allowed enough working distance for wingbeats under the microscope. Images were acquired at a frame rate of 98 Hz with 16 bit image depth at a 512 × 512 pixel spatial resolution. Myoplasmic calcium responses in DLM fibres were imaged in vivo through the dorsal cuticle either during tethered flight, or after electrical stimulation of selected MNs (Supplementary Video 4 shows myoplasmic calcium responses of DLM fibre 6 in response to electrical stimulation of the MN5 that innervates DLM fibres 5 and 6; Fig. 1a). Myoplasmic calcium signals were acquired using Hamamatsu photonics software (TOKUPIC v.1.0) and presented as percentage change (∆*F*/*F*) as previously described^21^.

### Software

Figures were created in Corel DRAW 2021 and Adobe Illustrator 2021. Acquisition for electrophysiology was conducted with pClamp (v.10.7) and data were further analysed using Spike2 (v.7.2) and GraphPad Prism (v.9.2.0). Calcium imaging data were acquired using Hamamatsu photonics software (TOKUPIC v.1.0). Model simulations were performed using the brian2 package (version 2.4.2.) for python^53^. The bifurcation analysis of the model was carried out using AUTO-07P^54^. Statistical tests were performed using scipy.stats of SciPy (v.1.7.1).

### Computational modelling and equations

#### Conductance-based MN model and gap junctional circuit model

Individual MNs are described by a single-compartment model with spike generating Na^+^ and K^+^ currents, *I*_Na_ and *I*_K_, based on ref. ^39^ and further refined on the basis of in situ current clamp and voltage clamp recordings from our laboratory (see below). For *I*_Na,_ the half-activation voltage of the activation gate is −33 mV, the half-activation voltage of the inactivation gate is −39.14 mV and the reversal potential *E*_Na_ is 55 mV. For *I*_K_ (referred to as *I*_shab_ in the equations below) the half-activation voltage is −42.14 mV and the reversal potential *E*_K_ is −72 mV. The maximal sodium conductance *g*_Na_ is 0.4312 µS and the maximal potassium conductance *g*_shab_ is varied between 0.13768216 µS and 0.34496 µS throughout the paper (see the ‘Shab-induced bifurcations’ section; a list of single-neuron-model parameters is provided in Supplementary Table 2). For the gap junctional circuit model, identical single-neuron models were coupled by linear non-rectifying gap junction currents, *I*_gap,_ with properties based on dual patch current clamp recordings from MN pairs. The current–balance equation of the *i*^th^ neuron reads

$${C}_{{\mathtt{m}}}{\dot{v}}_{i}={I}_{{\rm{i}}{\rm{n}}}-{I}_{{\mathtt{L}}}({v}_{i})-{I}_{{\rm{s}}{\rm{h}}{\rm{a}}{\rm{b}}}({v}_{i})-{I}_{{\rm{N}}{\rm{a}}}({v}_{i})+\sum _{j\ne i}{I}_{{\rm{g}}{\rm{a}}{\rm{p}}}^{ij}({v}_{i},{v}_{j}).$$

where *v* is the membrane voltage and *C*_m_ is the membrane capacitance. The leak current *I*_L_ with the leak conductance *g*_L_, the potassium current *I*_shab_, and the sodium current *I*_Na_ are defined as 

$${I}_{{\mathtt{L}}}(v)={g}_{{\mathtt{L}}}(v-{E}_{{\rm{L}}}),$$

$${I}_{{\rm{s}}{\rm{h}}{\rm{a}}{\rm{b}}}(v)={g}_{{\rm{s}}{\rm{h}}{\rm{a}}{\rm{b}}}{b}^{4}(v-{E}_{{\mathtt{K}}}),$$

$${I}_{{\rm{Na}}}(v)={g}_{{\rm{Na}}}{m}_{{\rm{\infty }}}^{3}(v)(1-h)(v-{E}_{{\rm{Na}}}).$$

Gates of the voltage-dependent ion channels follow first-order kinetics

$${\tau }_{b}(v)\dot{b}={b}_{{\rm{\infty }}}(v)-b,$$

$${\tau }_{h}(v)\dot{h}={h}_{{\rm{\infty }}}(v)-h,$$

The bidirectional, non-rectifying gap junctions with linear charge transfer (Fig. 2e,f) are described by the following current

1 $${I}_{{\rm{gap}}}^{ij}\left({v}_{i},{v}_{j}\right)={g}_{{\rm{gap}}}^{ij}\left({v}_{j}-{v}_{i}\right).$$

Parameters common to all simulations are provided in Supplementary Table 2. For simulations with homogeneous coupling (Fig. 3b–d,j (left)) a coupling strength of ${g}_{{\rm{gap}}}^{ij}\,=\,43.5\,{\rm{pS}}$ for all *i* ≠ *j* was used. To keep the cases as comparable as possible, the mean coupling strength in the simulations with heterogeneous coupling (Fig. 3h,j) was chosen to be the same as in the homogeneous case. The ratios of coupling coefficients for MN1–4 were chosen to be the same as measured in vivo (Fig. 2f). MN5 was coupled in with a coupling strength half of the mean of the other connections. This yielded ${g}_{{\rm{gap}}}^{12}={g}_{{\rm{gap}}}^{21}={g}_{{\rm{gap}}}^{34}={g}_{{\rm{gap}}}^{43}=86.59\,{\rm{pS}}$, for the MN5 connections ${g}_{{\rm{gap}}}^{15}={g}_{{\rm{gap}}}^{51}={g}_{{\rm{gap}}}^{25}={g}_{{\rm{gap}}}^{52}={g}_{{\rm{gap}}}^{35}={g}_{{\rm{gap}}}^{53}=\,{g}_{{\rm{gap}}}^{45}={g}_{{\rm{gap}}}^{54}\,=$ 27.19 pS and 38.27 pS for the remaining connections. The coupling strength in the case of strong coupling (Fig. 3d) was chosen to be 3 nS for all connections.

Stochastic and deterministic simulations of the model were performed using the brian2 package (v.2.4.2) for Python^53^. For stochastic simulations, the Heun method was used with a time step of 3 µs. For noisy simulations, a zero-mean white-noise current was added to the current balance equation (using the brian2 xi variable) with a noise strength of $\sigma =0.949\,{\rm{pA}}\sqrt{{\rm{ms}}}$ (Fig. 3d,h,j). For the deterministic simulations in Fig. 3b,c, the classical Runge–Kutta method (RK4) was used with a time step of 100 µs.

In addition, *Q* represents $\frac{e}{{k}_{{\rm{B}}}T}$, which is the elementary charge *e* divided by the Boltzmann constant *k*_B_ and the temperature *T* in Kelvin. Throughout the simulations, *Q* was approximated as 39.2/*V*.

The model parameters common to all models are summarized in Supplementary Table 2; the activation curves of the gates and the kinetic time scales are provided in Supplementary Table 3. Some parameters and equations were reported inconsistently in the original publication. Because the authors^39^ provided their code, we were able to extract the parameters used for their simulations, which are reported here. These updated parameters were validated using additional current-clamp and voltage-clamp recordings from our laboratory. Furthermore, to produce different onset bifurcations, the Shab conductance was changed (see below) and the input current was adjusted to move the system close to onset. The values differing between models are summarized in Supplementary Table 4.

For almost all simulations, neurons were initiated in random phases, by drawing timepoints from within one ISI and initializing the simulation with the corresponding state vector of a periodically spiking uncoupled neuron. For the simulations in Fig. 3c, the same initial conditions were used for each coupling strength. For the example in Fig. 3b, the initial conditions were not random, but hand-picked to yield a sequence that matches one of the preferred sequences in Fig. 1f.

Gap junctions that adhere to equation (1) affect the intrinsic properties of neurons because they act as effective leak and capacitance changes. For example, gap-junction currents can influence firing frequencies depending on the synchronization state of the network. In Fig. 3c, the coupling is homogeneous ${g}_{{\rm{gap}}}^{i,j}={g}_{{\rm{gap}}}$ for all *i* ≠ *j* between pairs but the coupling strength *g*_gap_ is varied. The effect of coupling on firing frequencies is strongest in the intermediate coupling range. In the weak coupling range, the effect on frequency is small because the current flow through gap junctions is small. In the strong coupling range, the neurons synchronize and, consequently, almost no gap-junctional current flow occurs. As frequency homeostasis is not part of this model, we accept biologically implausible firing frequencies in the intermediate range. In biological neurons, a wide range of mechanisms—for example, based on additional ion channels, energy consumption or ionic concentrations—could stabilize the frequencies^55^.

#### Shab-induced bifurcations

A local bifurcation analysis of the fixpoints in the model was shown previously^39^. The important quantity for predicting network states of the CPG is the PRC (Fig. 3e), which is closely related to the spike-onset bifurcation of the neuron and, therefore, the neuron’s excitability class. The bifurcation diagram in Extended Data Fig. 9a adds non-local and codim-2 bifurcations that organize transitions between excitability classes and their PRCs such as big SNL and small SNL^37,56^. Depending on the Shab channel density, *g*_shab_ on the ordinate, different onset bifurcations that change the resting state into the spiking mode occur. In the middle range of *g*_shab_ levels, spiking commences through a well-known SNIC bifurcation. The SNIC region is encapsulated by two SNL points. At the lower end, for *g*_shab_ smaller than the small SNL (sSNL) point, spiking commences through a small homoclinic loop (HOM, green line in Extended Data Fig. 9a) at lower input currents, *I*_in_, than the saddle-node bifurcation (blue line in Extended Data Fig. 9a). At the upper end of the SNIC range, a series of bifurcations is traversed that eventually leads to spike onset through a fold of limit cycles (FLC, bifurcation line not shown) and a subsequent subcritical Hopf bifurcation, here termed the Hopf regime for simplicity. Bifurcations leading from SNIC to the Hopf excitability class are the big SNL, the neutral saddle loop (not shown) and also a cusp^56^. For simplicity, in the main text, when using the term SNL, we refer to sSNL. The Bogdanov–Takens point in between creates an Adronov–Hopf line (red line in Extended Data Fig. 9a) that eventually turns back to create the excitation block at higher input levels.

The consequences for the phase relationships of coupled neurons can be understood from the neuron’s PRC at different bifurcations (Extended Data Fig. 9a). A neuron’s ability to produce arbitrarily low firing rates depends on its excitability class (determined by the spike onset bifurcation). So does the PRC shape^57–59^. The insets in Extended Data Fig. 9a show prototypical PRCs for the three onset bifurcations, which can be achieved at different levels of Shab-channel density. The exact parameters are summarized in Supplementary Table 4. A critical transition with repercussions for the CPG network states is the symmetry breaking in the PRC at the sSNL bifurcation as described in the next section^59^.

An FLC/Hopf onset bifurcation as spike onset can be ruled out as a model for the biological MN1–5 network based on the following grounds: the region in which tonic spiking commences through the FLC/Hopf bifurcation has a finite, non-zero frequency at the rheobase. For the present model (Extended Data Fig. 9a (top arrow)), this is at around 47 Hz and therefore above the dynamic range of the in vivo measures *f*–*I* curves from the MNs, which operates between 0–40 Hz (3—15 Hz during normal flight; Fig. 1e).

The bifurcation analysis was performed using AUTO-07P^54^.

#### Mechanism of splay state generation

Our theoretical analysis predicts that dynamics close to the SNL point favour splay states^59^. This prediction is based on coupling functions, which are obtained from the biophysical conductance-based models using a phase-reduction (see the next section).

The coupling function *G*(*ψ*) combines a neuron’s PRC and a synaptic transfer term, which, for the system at hand, captures the voltage perturbations caused by gap-junctional coupling (equation (4)). The coupling function describes how one neuron’s phase is shifted based on the phase difference to another neuron. Of particular interest is the odd part of the coupling function, *G*^odd^(*ψ*) = *G*(*ψ*) − *G*(−*ψ*). It reflects the reciprocity of coupling between two neurons and extracts how coupling affects the phase difference in the cells’ firing. Phase relationships of tonically firing neurons can be read from *G*^odd^ (Fig. 3e (bottom)).

Crucially, stable phase relationships depend on a stable fix point in this odd part of the coupling function, that is, a zero crossing with a negative slope. The shape of the coupling function in turn depends on the bifurcation type of the neuron (Extended Data Fig. 9a). For example, for a PRC of a neuron with SNIC dynamics, a small phase distance, Δ*φ*, (neuron 1 fires shortly before neuron 2) is further decreased, whereas a large Δ*φ* (neuron 2 fires shortly before neuron 1) is further increased. This results in a stable fixpoint at phase 0, so that the neurons show synchronized firing (Fig. 3e (bottom)). By contrast, with a PRC at the SNL point, a small Δ*φ* is increased, whereas a large Δ*φ* is decreased. This results in a stable fixpoint at Δ*φ* = 0.5 for each coupled neuron pair. For a network of >2 electrically coupled neurons, this causes a frustrated state because antiphase locking at Δ*φ* = 0.5 cannot be achieved for all units at the same time. In a small network, such as the MN1–5 network with its five coupled neurons, the splay state represents a low-frustration solution that determines the most likely network state^60^.

These analytical predictions based on coupling functions were corroborated by simulations of the full biophysical model. Near the SNL point, networks of five gap-junction-coupled neurons indeed showed a splay state (Fig. 3b), whereas neurons with a SNIC onset synchronized instead (Fig. 3h). These simulations show that, for our parameters, the phase reduction is valid and that coupling functions based on the phase reduction accurately capture the network’s synchronization behaviour. On the basis of both coupling functions and simulations, we predict that the combination of coupling and intrinsic excitability determines the splay state in the biological MN1–5 network.

#### Phase-reduced circuit model with repulsive coupling

To generate coupling functions, we reduce the biophysical, conductance-based neurons to approximate phase oscillators^61^. This is warranted because the cholinergic inputs drive MN1–5 into a tonically spiking regime. The *i*^th^ phase oscillator is defined by its intrinsic, mean firing rate, *f*_*i*_, and its phase response curve, *Z*(*φ*_*i*_), obtained from the biophysical model (Extended Data Fig. 9a). The PRCs are calculated based on a direct perturbation approach^62,63^. The network equation reads:

2 $${\dot{{\phi }}}_{i}={f}_{i}+\sum _{j\ne i}Z({{\phi }}_{i})g({{\phi }}_{i},{\phi }_{j}),$$

where *g*(*φ*_*i*_,*φ*_*j*_) is the phase-dependent perturbation received through the gap junctions. It depends on the phases of the presynaptic and postsynaptic neurons. If the spike waveforms are uniform, the interaction term, based on equation (1) reads

$$g({{\phi }}_{i},{{\phi }}_{j})={g}_{{\rm{g}}{\rm{a}}{\rm{p}}}(v({{\phi }}_{j})-v({{\phi }}_{i}))/{C}_{{\rm{m}}}.$$

To understand the influence of the PRC on phase relationships in small networks, first consider the phase difference between two coupled neurons *i* and *j*, isolated from the network. Define their phase difference *ψ* = *φ*_*i*_ − *φ*_*j*_. Then, using approximate averaging theory, the slow evolution of the phase difference simplifies^33^ to

3 $$\dot{\psi }=\nu +{G}^{{\rm{odd}}}\left(\psi \right).$$

Here, the small frequency detuning is *ν* = *f*_*i*_ − *f*_*j*_ and the averaged coupling function is defined as

4 $$G(\psi )={\int }_{0}^{1}Z({{\phi }}_{i})g({{\phi }}_{i},{{\phi }}_{i}-\psi )d{{\phi }}_{i}.$$

Stable fixpoints of equation (3) determine constant phase relations for neurons with similar intrinsic frequencies as illustrated in Extended Data Fig. 9b,c. In Fig. 3e the odd part, *G*^odd^(*ψ*), and the fixpoints are shown for different choices of *Z*(*φ*). The stable in-phase fixpoint directly translates to a synchronous network state also for network sizes of *N* > 2. However, this is not observed in the CPG recordings (Fig. 1a). For the network to show frustration, pairs of neurons must be phase-repellent.

The top spike trains in Extended Data Fig. 9b illustrate an example that generates stable antiphase fixpoints. To explain stable phase relationships graphically, the model was simplified, such that the shape of the coupling is a scaled version of the PRC (corresponding to gap junction coupling with delta spikes). Moreover, the mean PRC was absorbed into the average firing rate. The stability can be analysed using the iterated map of the phase differences. The return map reads *ψ*_*n*+1_ = *ψ*_*n*_ + *Z*^odd^(*ψ*_*n*_). Extended Data Fig. 9c uses iterated maps to show how a PRC with a negative slope at *φ* = 0.5 leads to a stable fixpoint in antiphase (left). An example with a positive slope at *φ* = 0.5 is shown in Extended Data Fig. 9b (bottom) and Extended Data Fig. 9c (right) and shows in-phase synchronization.

In conclusion, reciprocally, yet weakly coupled neurons, such as those connected through non-rectifying gap junctions, require an asymmetric coupling function (that is, with a substantial odd part) to show stable phase locking. For antiphase synchronization to be stable and the network in equation (2) to show frustration, the odd part of the coupling function must have a stable fixpoint at phase 0.5. In the low-frequency limit, this can be achieved with a PRC that has a negative slope at *φ* = 0.5, such as the PRC at the SNL point.

#### Calculating model coupling coefficients

Coupling coefficients for the model were calculated analytically. For this, we looked at pairs of gap-junction-coupled neurons (not the whole network of five neurons) and it was assumed that the voltage is sufficiently negative for all voltage-gated ion channels to be closed. In this case, the relationship between coupling coefficient (CC) and gap junction strength (*g*_gap_) reads

$${\rm{C}}{\rm{C}}=\frac{{g}_{{\rm{g}}{\rm{a}}{\rm{p}}}}{{g}_{{\rm{g}}{\rm{a}}{\rm{p}}}+{g}_{{\mathtt{L}}}}.$$

This relationship was verified in simulations that mimicked the experimental approach to determine coupling coefficients^29^. Specifically, two neurons coupled through gap junctions received steady current inputs below the firing threshold. The first neuron, n_1_, received a baseline current *I*_base_ = −150 pA, the other neuron, n_2_, received an additional perturbation current *I*_base_ + Δ*I* (Δ*Ι* from −5 pA to 5 pA). A third, uncoupled neuron n_3_ was simulated with *I*_base_ input. Simulations were run until all neurons reached steady state, resulting in steady state voltages *v*_1_, *v*_2_ and *v*_3_. Then, the coupling coefficients were calculated as ${\rm{CC}}=\frac{{v}_{1}-{v}_{3}}{{v}_{2}-{v}_{3}}$. Here, a low *I*_base_ ensures that the resulting CC is steady over the range of Δ*Ι* currents and matches the analytical results.

#### The splayness index

When spike times of all *N* neurons in a network are available, splayness can be quantified by comparing the neuron’s phase differences to those that would arise in a perfect splay state.

To this end, we first discretized the time from the first spike of the last neuron that started spiking until the last spike of the first neuron that stopped spiking with a time step of 1 ms. If there were breaks in the recording (where the fly stopped and started flying again), those times were omitted. Again, the breaks were defined from the last spike of the first neuron that stopped spiking until the first spike of the last neuron that started spiking. This way, all phases of all *N* neurons were sampled with a 1 ms time step at *K* time points *τ*_*k*_ = *k* ms for *k* = 0, 1, ..., *K* − 1.

For each neuron *i*, its phases were interpolated between its spikes *t*_*n,i*_ as:

5 $${{\phi }}_{k,i}=\frac{{\tau }_{k}-{t}_{n,i}}{{t}_{n+1,i}-{t}_{n,i}},({t}_{n,i}\le {\tau }_{k} < {t}_{n+1,i})\,{\rm{f}}{\rm{o}}{\rm{r}}\,i=1,2,\ldots \,N.$$

Then, for each point in time, the phases were ordered such that

$${{\phi }}_{k,l} > {{\phi }}_{k,m},(l > m).$$

Phase differences were computed as

$${\psi }_{k,i}={{{\phi }}_{k,i+1}-{\phi }}_{k,i}\,{\rm{f}}{\rm{o}}{\rm{r}}\,i=1,...N-1$$

and

$${\psi }_{k,N}=1-\mathop{\sum }\limits_{i=1}^{N-1}{\psi }_{k,i}.$$

To calculate the splayness, these phase differences were compared to the phase differences of the most splayed state (splay state) and to those of the least splayed state (sync state):

6 $${\gamma }_{k}=\frac{\mathop{\sum }\limits_{i=1}^{N}{\left({\psi }_{k,i}-\frac{1}{N}\right)}^{2}}{(N-1){N}^{-2}+{(1-{N}^{-1})}^{2}}=\frac{N}{N-1}\mathop{\sum }\limits_{i=1}^{N}{\left({\psi }_{k,i}-\frac{1}{N}\right)}^{2}.$$

From this we constructed the time-averaged splayness

7 $$s=1-\sqrt{\frac{1}{K}\sum _{k}{\gamma }_{k}}.$$

With this definition, a perfectly splayed network yields *s* = 1 and a perfectly in-phase synchronized network yields *s* = 0.

#### Analysing pairs of neurons (synchronization index and phase histogram dips)

Many experimental recordings include only two of the five MNs (Fig. 3d,h). In these cases, the splayness index cannot be used. Here we relied on a pairwise synchronization measure instead. Specifically, we interpolated the phases of the two neurons in question according to equation (5). Then, a classical Kuramoto order parameter was evaluated:

$${r}_{k}=| \frac{1}{2}\mathop{\sum }\limits_{j=1}^{2}{{\rm{e}}}^{i2{\rm{\pi }}{{\phi }}_{k,j}}| .$$

Its time average $r=\frac{1}{K}{\sum }_{k}{r}_{k}$ measures in-phase synchronization of the neuron pair, where *K* is the number of time points.

A low synchronization index does not necessarily imply a splay state, as there are other desynchronized network states with equally low synchronization values. We therefore investigated alternative ways to gain information about splayness in the full MN1–5 network when in vivo recordings only comprised two neurons. The shape of pairwise phase histograms provides such information. Specifically, we observed that networks with high splayness often display a dip in pairwise histograms around zero. To test whether this dip is a good indicator for splayness, additional simulations were performed. With increasing noise strength, the dip around zero decreases, but it is more persistent than other histogram characteristics of splayed-out networks (Extended Data Fig. 6a). Furthermore, there was a strong negative correlation between the fraction of the MN4–MN5 pairwise histogram within (−0.1, 0.1) and the full-network splayness in experimental recordings for which all five neurons were available (Extended Data Fig. 6d). Together, this led us to conclude that the dip around zero is an appropriate indicator for splayness.

#### Comparing synchronization indices

To compare synchronization indices (Fig. 3d,h), Mann–Whitney *U*-tests were performed using scipy.stats of SciPy v.1.7.1. Tests were uncorrected because only a few comparisons based on our hypotheses were performed.

Regarding the results of Fig. 3d, MN4–MN5 synchronization indices for simulations with SNL onset and strong coupling (Mdn = 1.0) were higher than those for simulations with SNL onset and weak coupling (Mdn = 0.54). A two-sided Mann–Whitney *U*-test indicated that this difference was statistically significant, *U*(*N*_strong SNL_ = 10, *N*_weak SNL_ = 10) = 100, *P* = 0.0002. MN4–MN5 synchronization indices for flies with *shakB* RNAi (Mdn = 0.61) were higher than those for control animals (Mdn = 0.56). A two-sided Mann–Whitney *U*-test indicated that this difference was statistically significant, *U*(*N*_shakB-RNAi_ = 9, *N*_control_ = 11) = 78, *P* = 0.0334. MN4–MN5 synchronization indices for flies with *shakB* overexpression (Mdn = 0.84) were higher than those for control animals (Mdn = 0.56). A two-sided Mann–Whitney *U*-test indicated that this difference was statistically significant, *U*(*N*_shakB-OE_ = 7, *N*_control_ = 11) = 73, *P* = 0.0008.

Regarding the results of Fig. 3h, MN3–MN4 synchronization indices for SNIC simulations (Mdn = 0.99) were higher than those for SNL simulations (Mdn = 0.26). A two-sided Mann–Whitney *U*-test indicated that this difference was statistically significant, *U*(*N*_SNIC_ = 10, *N*_SNL_ = 10) = 100, *P* = 0.0002. MN3–MN4 synchronization indices for flies with *Shab* overexpression (Mdn = 0.52) were higher than those for control flies (Mdn = 0.43). A two-sided Mann–Whitney *U*-test indicated that this difference was statistically significant, *U*(*N*_shab-OE_ = 10, *N*_control_ = 8) = 63, *P* = 0.0434.

### Reporting summary

Further information on research design is available in the Nature Portfolio Reporting Summary linked to this article.

## Online content

Any methods, additional references, Nature Portfolio reporting summaries, source data, extended data, supplementary information, acknowledgements, peer review information; details of author contributions and competing interests; and statements of data and code availability are available at 10.1038/s41586-023-06099-0.

## Supplementary information

Supplementary Information Supplementary Tables 1–5, listing reagents for experiments, parameters for computational modelling and the genotypes of all of the fly strains used.

Reporting Summary

Supplementary Video 1 Video *of Drosophila melanogaster* during tethered flight. High-speed video (5,000 fps) of *D. melanogaster* during tethered flight with one recording electrode.

Supplementary Video 2 Video of *Lucilia* sp. during tethered flight. High-speed video (5,000 fps) of *Lucilia* sp. during tethered flight with 2 recording electrodes.

Supplementary Video 3 Video of *A. mellifera* during tethered flight. High-speed video (5,000 fps) of *A. mellifera* during tethered flight.

Supplementary Video 4 In vivo calcium imaging from the DLM fibres of *D. melanogaster.* In vivo calcium imaging from DLM fibre 6 with the expression of GCaMP8f (w[*];P{y[+t7.7] w[+mC]=20XUAS-IVS-jGCaMP8f}su(Hw)attP5/+;P{[+mC]=Act88F-GAL4.1.3}3/+) in response to electrical stimulation with different frequencies (0.5, 1, 2, 5 and 10 Hz). The field of view contains the dorsal thorax with the scutellum (purple line) at the posterior end and the head anteriorly (out of focus). The DLM fibre on one side is encircled (cyan) and the region of interest for ∆*F*/*F* GCaMP8f intensity measurements is indicated by a green circle. Calcium responses of the stimulated DLM fibre are shown as a video in real time, and ∆*F*/*F* values are played synchronously on the top.

## Data Availability

The electrophysiology and imaging data have been deposited at the Zenodo open data repository (10.5281/zenodo.7737730). Computational modelling data and additional computational analyses of in vivo electrophysiological data have been deposited at the Zenodo open data repository (10.5281/zenodo.7740678).

## Code availability

Code for implementing the computational model and for electrophysiological data analyses is available at Zenodo (10.5281/zenodo.7740678).
