# Supplementary material for: Gap junctions desynchronize a neural circuit to stabilize insect flight
Source: Nature. 2023 May 24;618(7963):118–25. doi: 10.1038/s41586-023-06099-0 (PMC10232364; doi:10.1038/s41586-023-06099-0)
Supplement: Supplementary file 1 — Supplementary Tables 1–5, listing reagents for experiments, parameters for computational modelling and the genotypes of all of the fly strains used. [file 41586_2023_6099_MOESM1_ESM.pdf]

nature portfolio

https://doi.org/10.1038/s41586-023-06099-0

Supplementary information

Gap junctions desynchronize a neural circuit to stabilize insect flight

In the format provided by the authors and unedited

Supplementary information for

Gap junctions desynchronize a neural circuit to stabilize insect flight

This PDF file includes:

Supplementary Tables 1 to 5

Other Supplementary Materials for this manuscript include the following:

Supplementary Videos 1 to 4
SI guide with captions for Supplementary Videos 1 to 4

Supplementary Tables

Table 1: Reagents.

REAGENT or RESOURCE	SOURCE	IDENTIFIER	
Chemicals, Peptides, and Recombinant Proteins			
Streptavidin Alexa 647	Thermo Fisher Scientific, Cat# S-21374	RRID:AB_2336066	
TRITC-Tetramethylrhodamine Dextran 3000 lysin fixable	Thermo Fisher Scientific, Cat# 3308		
Neurobiotin Tracer	Vector Labs, Cat# SP-1120	RRID:AB_2336606	
Chicken Anti-GFP primary antibody, polyclonal	Thermo Fisher, Cat# A10262	RRID: AB_2534023	
Alexa Fluor 488 AffiniPure Donkey Anti-Chicken IgY (IgG) (H+L)	Jackson Immunoresearch Cat# 703-545-155	RRID: AB_2340375	
Rat anti-mCherry monoclonal Antibody (16D7)	Thermo Fisher Scientific, Cat #M11217	RRID:AB_2536611	
Alexa Fluor 594-AffiniPure Fab Fragment Donkey Anti-Rat IgG (H+L)	Jackson Immunoresearch Cat# 712-587-003	RRID:AB_2340692	
Tetrodotoxin citrate	Bio-Techne, Cat# 1069		
TritonX-100	Sigma Aldrich, Cat# T8787; CAS 9002-93-1		
Protease Type XIV from *Streptomyces griseus*	Sigma Aldrich, Cat# P5147; CAS 9036-06-0		
Sodium nitrite	Sigma Aldrich, Cat# 563218; CAS 7632-00-0		
Potassium hydroxide	Sigma Aldrich, Cat# 306568, CAS 1310-58-3		
Methylsalicylate	Sigma Aldrich, Cat# M6752; CAS 119-36-8		
Sylgard 184 Dow Corning	VWR Chemicals, Cat# 634165S		
Experimental Models: Organisms/Strains			used in Figure
D. melanogaster: wildtype strain Canton Special (Canton S)	lab culture	RRID:BDSC_64349	Fig. 1<br>Fig. 2<br>Fig. 3<br>ED Fig. 1<br>ED Fig. 2<br>ED Fig. 4<br>ED Fig. 8

D. melanogaster: *ShakB*^{RNAi} in DLM MNs<br>$\frac{w}{\rightharpoondown};\frac{GMR23H06-pBPp65ADZpUw\}attp40}{+};\frac{GMR30A07-pBPZpGAL4.BD.Uw\}attP2}{P\{TRiP.HMC04895\}attP2}$	the GMR23H06-ADZ;30A07-DBD DLM Split GAL4 stock was made in our own lab with plasmids as described in the HHMI Janelia Farm Research Campus Fly Light Collection by G. Rubin (Jenett et al. 2012)	RRID:<br>BDSC_57706	Fig. 2a,b,g<br>Fig. 3
D. melanogaster: ShakB^{OE} in DLM MNs<br>$\frac{w}{\rightharpoondown};\frac{GMR23H06-pBPp65ADZpUw\}attp40}{UAS-shakb(N+16)};\frac{GMR30A07-pBPZpGAL4.BD.Uw\}attP2}{+}$	DLM Split GAL4	RRID:<br>BDSC_86262	Fig. 2a,b<br>Fig. 3d
D. melanogaster: ShakB^{KO} in DLM MNs<br>$\frac{w[1118]}{\rightharpoondown};\frac{P\{TKO.GS01152\}attP40}{+};\frac{P\{UAS-Cas9.C\}attP2}{P\{GMR23H06-GAL4\}attP2}$	Stock BDSC_49050 was discontinued	RRID:<br>BDSC_78593<br>BDSC_54595<br>BDSC_49050	
D. melanogaster: control for vermilion-based transgenes (e.g. *RNAi)* in DLM MNs<br>$\frac{w}{\rightharpoondown};\frac{GMR23H06-pBPp65ADZpUw\}attp40}{+};\frac{GMR30A07-pBPZpGAL4.BD.Uw\}attP2}{P\{UAS-GFP.VALIUM10\}attP2}$	DLM Split GAL4	RRID:<br>BDSC_35786	Fig. 1<br>Fig. 2<br>Fig. 3<br>ED Fig. 1<br>ED Fig. 2<br>ED Fig. 4a,b<br>ED Fig. 8
D. melanogaster: control for mini white-based transgenes (e.g. *RNAi or overexpression)* in DLM MNs<br>$\frac{w}{\rightharpoondown};\frac{GMR23H06-pBPp65ADZpUw\}attp40}{+};\frac{GMR30A07-pBPZpGAL4.BD.Uw\}attP2}{+}$	DLM Split GAL4	RRID:<br>BDSC_35786	Fig. 2<br>Fig. 3f<br>ED Fig. 1<br>ED Fig. 2<br>ED Fig. 3<br>ED Fig. 5<br>ED Fig. 6e,f<br>ED Fig. 10
D. melanogaster: *GluClα*^{RNAi} mainly in DLM MNs<br>$\frac{w[1118]}{\rightharpoondown};;\frac{P\{KK109167\}VIE-260B}{P\{GMR23H06-GAL4\}attP2}$	Stock BDSC_49050 was discontinued	RRID:<br>FlyBase_FBst0477580<br>BDSC_49050	ED Fig. 6e
D. melanogaster: *Rdl*^{RNAi} mainly in DLM MNs<br>$\frac{w[1118]}{\rightharpoondown};;\frac{P\{GD4609\}v41103}{P\{GMR23H06-GAL4\}attP2}$	Stock BDSC_49050 was discontinued	RRID:<br>FlyBase_FBst0463935<br>BDSC_49050	ED Fig. 6f
D. melanogaster: Channelrhodopsin XXL in DLM MNs<br>$\frac{w[1118]}{\rightharpoondown};\frac{GMR23H06-pBPp65ADZpUw\}attp40}{P\{UAS-ChR2.XXL\}VK00018};$<br>$\frac{GMR30A07-pBPZpGAL4.BD.Uw\}attP2}{+}$	DLM Split GAL4	RRID:<br>BDSC_58374	ED Fig. 5c-e
D. melanogaster: Channelrhodopsin XXL in cholinergic neurons<br>$\frac{w[1118]}{\rightharpoondown};\frac{P\{UAS-ChR2.XXL\}VK00018}{P\{w[+mC]=ChAT-GAL4.7.4\}19B}$		RRID:<br>BDSC_6798<br>BDSC_58374	ED Fig. 3

D. melanogaster: *FMRP*^{RNAi} in GFP-expressing DLM MNs<br>$\frac{w}{\rightharpoondown};\frac{P\{GMR23H06-ADZ\}attp40\ P\{UAS-CD4-tdGFP\}8M2}{P\{KK107935\}VIE-260B};$<br>$\frac{P\{GMR30A07-DBD\}attP2}{+}$	DLM Split GAL4	RRID:<br>BDSC_35839<br>FlyBase_FBst0482375	Fig. 2c
D. melanogaster: fast GCaMP8f expression in DLM muscle<br>$\frac{w}{\rightharpoondown};\frac{P\{20XUAS-IVS-jGCaMP8f\}su(Hw)attP5}{+};\frac{P\{Act88F-GAL4.1.3\}3}{+}$	Zhang et al., 2023, Nature 615(7954): 884-891	RRID:<br>BDSC_38461<br>BDSC_92588	Fig. 4b,c
D. melanogaster: trans-tango in DLM MNs<br>$\frac{y^1w^*\ \mathrm{UAS-myrGFP.QUAS-mtdTomato-3xHA\}su(Hw)attP8}}{w^*};$<br>$\frac{GMR23H06-pBPp65ADZpUw\}attp40}{\mathrm{P\{y[+t7.7]\ w[+mC]=trans-Tango\}attP40}};\frac{GMR30A07-pBPZpGAL4.BD.Uw\}attP2}{+}$	Talay et al., 2017, Neuron 96(4): 783--795.e4	RRID:<br>BDSC_77124	ED Fig. 7c-cii
D. melanogaster: trans-tango in period-interneurons<br>$\frac{y^1w^*\ \mathrm{UAS-myrGFP.QUAS-mtdTomato-3xHA\}su(Hw)attP8}}{w^*};$<br>$\frac{P\{w[+mC]=GAL4-per.BS\}3}{\mathrm{P\{y[+t7.7]\ w[+mC]=trans-Tango\}attP40}};\frac{+}{+}$	Talay et al., 2017, Neuron 96(4): 783--795.e4	RRID:<br>BDSC_77124<br>BDSC_7127	ED Fig. 7b-bii
D. melanogaster: trans-tango in LC4 visual interneurons<br>$\frac{y^1w^*\ \mathrm{UAS-myrGFP.QUAS-mtdTomato-3xHA\}su(Hw)attP8}}{w^*};$<br>$\frac{P\{y[+t7.7]w[+mC]=R47H03-p65.AD\}attP40}{\mathrm{P\{y[+t7.7]w[+mC]=trans-Tango\}attP40}};$<br>$\frac{P\{y[+t7.7]\ w[+mC]=R86D05-GAL4.DBD\}attP2}{+}$	Talay et al., 2017, Neuron 96(4): 783--795.e4<br><br>Janelia Farm LC4_2-Split GAL4, Stock SS00356	RRID:<br>BDSC_77124	ED Fig. 7a-aii
D. melanogaster: active zone marker bruchpilot short to mark output synapses<br>$\frac{w^*}{\rightharpoondown};\frac{UAS-brp.S(D3)-mStrawberry}{GMR23H06-pBPp65ADZpUw\}attp40\quad P\{UAS-CD4-tdGFP\}8M1};$<br>$\frac{GMR30A07-pBPZpGAL4.BD.Uw\}attP2}{+}$	S. Sigrist, Free University of Berlin	RRID:<br>BDSC_35839	ED Fig. 7d-dii,<br>ED Fig. 7e-eii
D. melanogaster: overexpression (oe) of Shab delayed rectifier K$^+$ channel:<br>$\frac{w^*}{\rightharpoondown};\frac{+}{GMR23H06-pBPp65ADZpUw\}attp40};\frac{GMR30A07-pBPZpGAL4.BD.Uw\}attP2}{P\{UAS-Shab\}pJFRC81\ attP2}$	*UAS-Shab* fly strain was generated in this study		Fig. 3f,g,h<br>ED Fig. 10
D. melanogaster: *Shab*^{RNAi} in DLM MNs<br>$\frac{w^*}{\rightharpoondown};\frac{P\{UAS-TRiP.HMS02400\}attP40}{GMR23H06-pBPp65ADZpUw\}attp40};\frac{GMR30A07-pBPZpGAL4.BD.Uw\}attP2}{+}$	Perkins et al., 2015, Genetics 201(3): 843--852	RRID:<br>BDSC_41999	ED Fig. 10
D. melanogaster: activity-dependent GRASP between cholinergic interneurons and DLM MNs<br>$\frac{w^*}{\rightharpoondown};\frac{P\{w[+mC]=lexAop-nSyb-spGFP1-10\}2,P\{w[+mC]=UAS-CD4-spGFP11\}2}{GMR23H06-pBPp65ADZpUw\}attp40\ P\{w[+mC]=UAS-CD4-tdTom\}7M1};$<br>$\frac{GMR30A07-pBPZpGAL4.BD.Uw\}attP2}{TI\{2A-lexA::GAD\}ChAT[2A-lexA]}$	DLM Split GAL4	RRID:<br>BDSC_64315<br>BDSC_84379<br>BDSC_35841	ED Fig. 4a,b

D. melanogaster: expression of membrane tethered tandem GFP in DLM MNs $\frac{w^*}{\rightharpoondown}; \frac{GMR23H06-pBPp65ADZpUw\}attp40 \;\; P\{UAS-CD4-tdGFP\}8M1}{+}; \frac{GMR30A07-pBPZpGAL4.BD.Uw\}attP2}{+}$	DLM Split GAL4	RRID: BDSC_35839	ED Fig. 5a,b
Lucilia spec. (gold fly)	md-terraristik.de		ED Fig. 2
Drosophila hydei	local pet shop		ED Fig. 2
Calliphora spec. (blowfly)	home-of-insects.com		ED Fig. 2
Musca domestica (house fly)	local pet shop		ED Fig. 2
Apis mellifera (honey bee)	local apiculture of University of Mainz		ED Fig. 2
Software and Algorithms			
Amira 4.1 with custom plug-ins	FEI Hillsboro, Oregon, US; Schmitt el al. 2004, Evers et al. 2005	Amira 3D analysis , RRID:SCR_014305	
Corel Draw 2021	Corel Corporation	RRID:SCR_014235	
SPSS Statistics 22	IBM	SPSS, RRID:SCR_002865	
Huygens Professional software (version 19.10.0)	Scientific Volume Imaging	RRID:SCR_014237	
IR illuminator with 48 LEDs	Sygonix		
pClamp10.7 electrophysiology software	Molecular Devices	RRID:SCR_011323	
Graphpad Prism 9.2.0	GraphPad Software, San Diego, USA	RRID:SCR_002798	
Spike2 (Version 7.2)	CED	RRID:SCR_000903	
FASTCAM Viewer software (PFV Version 3.6.9.0)	Photron		
Leica Application Suite X (LASX)	Leica Microsystems	RRID:SCR_013673	
Illustrator 2021 version 25.2	Adobe	RRID:SCR_010279	
Jupyter Notebook (custom Python routines)		RRID:SCR_018315	
Python library NumPy		RRID:SCR_008633	
Python library pickle			
Python library SciPy		RRID:SCR_008058	
Python library Matplotlib		RRID:SCR_008624	
Other			
Glass microelectrodes with filament, i.d. 0.5 mm, o.d. 1.0 mm	Sutter Instruments, Cat# BF 100-50-10		
Dental Cromalux-E Halogen Curing Light Unit	Mega Physik, REF 7050.B01	REF 7050.B01	
clear glass adhesive	Duro; Pacer Technology, Rancho Cucamonga, CA		
Grass SD9 square pulse stimulator			
Sygonix IR illuminator with 48 LEDs			
Axopatch 200B Patch Clamp amplifier	Molecular Devices		
Digidata 1440 or 1550B analog/digital converter	Molecular Devices		
Patch glass without filament, i.d. 1.0 mm, o.d. 1.5 mm	World Precision Instruments, Cat# PG52151-4	Cat# PG52151-4	

PC-10 vertical pipette puller	Narishige		
P-97 Flaming Brown glass microelectrode puller	Sutter		
Osmomat 3000 basic freezing point osmometer	Gonotec		
Drosophila food vials, polystyrene, 68 ml	Kisker, Cat# 789008		
Model 1700 extracellular amplifier	AM-Systems		
FASTCAM Mini UX100	Photron		
tungsten rods, diameter 127 µm	Science Products, Cat# TW5-4	Cat# TW5-4	
tungsten wire, diameter 100 µm	MaTeck, Cat# 009486-1		
AxioExaminer A1 with Zeiss W Plan Apochromat 40x NA 1.0, DIC VIS-R lens	Zeiss, Germany		
TCS SP8 laser scanning confocal microscope with 40x oil objective	Leica Microsystems, Germany		

Table 2: Model parameters common to all models.

Parameter	Value
C_m	0.13 nF
g_{Na}	0.4312 μS
g_L	0.008624 μS
E_L	-60 mV
E_K	-72 mV
E_{Na}	55 mV
z_m	3
v_m	-33 mV
z_h	5.2
v_h	-39.14 mV
r_h	0.2/ms
γ_h	0.38
z_b	1.1056
v_b	-42.14 mV
r_b	0.2/ms
γ_b	0.38

Table 3: Activation functions and kinetic time scales of different gates.

gate	activation curve / kinetic timescale
p_∞	$\dfrac{1}{1+e^{-Qz_p(v-v_p)}}$
τ_p	$\dfrac{e^{-Qz_p\gamma_p(v-v_p)}}{r_p(1+e^{-Qz_p(v-v_p)})}$

Table 4: Model parameters differing between models.

Parameter	Hopf	SNIC	HOM/SNL
g_{shab}	0.34496 µS	0.2156 µS	0.13768216 µS
I_{in}	330 pA	175 pA	108.75 pA

Table 5: Primers for determination of the cloned Shab cDNA.

primer #	exons	Shab variants	sequence
22/23	2-9	A, B, J, L	ACGGGGACATCTATCCCACA (fw)<br>ATTCGCGCGCAATGGAATAC (rev)
24/25	2-8	C	ACGGGGACATCTATCCCACA (fw)<br>CGTGGAACACACAAATGCGA (rev)
26/27	2-4	K	GGTTGAGGCCGTGTGTATCA (fw)<br>CACTGGAGCTGTTCTGCTGT (rev)
28/29	10-13	H	AGTGCCAAAGCATCGGAAGA (fw)<br>GCTCTCCGGCGAGATTTAATG (rev)
30/31	5 long-10	I, N	GCGAGAGTCACACACGATCA (fw)<br>GTAGAGGGGCTGGTAGTGGA (rev)
32/33	3-10	M	CACCCCCAATATAGGCAGGC (fw)<br>TGGTAGTGGAGAGAGGAGCC (rev)
34/23	2-9	B	GTCGTGCATCCGAAGGGTAA (fw)<br>ATTCGCGCGCAATGGAATAC (rev)
