# Supplementary material for: Gap junctions desynchronize a neural circuit to stabilize insect flight
Source: Nature. 2023 May 24;618(7963):118–25. doi: 10.1038/s41586-023-06099-0 (PMC10232364; doi:10.1038/s41586-023-06099-0)
Supplement: Supplementary file 2 — Reporting Summary [file 41586_2023_6099_MOESM2_ESM.pdf]

nature portfolio

Corresponding author(s): Carsten Duch, Susanne Schreiber

Last updated by author(s): March 18, 2023

Reporting Summary

Nature Portfolio wishes to improve the reproducibility of the work that we publish. This form provides structure for consistency and transparency in reporting. For further information on Nature Portfolio policies, see our Editorial Policies and the Editorial Policy Checklist.

Statistics

For all statistical analyses, confirm that the following items are present in the figure legend, table legend, main text, or Methods section.

n/a	Confirmed	
☐	☒	The exact sample size (*n*) for each experimental group/condition, given as a discrete number and unit of measurement
☐	☒	A statement on whether measurements were taken from distinct samples or whether the same sample was measured repeatedly
☐	☒	The statistical test(s) used AND whether they are one- or two-sided<br>*Only common tests should be described solely by name; describe more complex techniques in the Methods section.*
☐	☒	A description of all covariates tested
☐	☒	A description of any assumptions or corrections, such as tests of normality and adjustment for multiple comparisons
☐	☒	A full description of the statistical parameters including central tendency (e.g. means) or other basic estimates (e.g. regression coefficient) AND variation (e.g. standard deviation) or associated estimates of uncertainty (e.g. confidence intervals)
☐	☒	For null hypothesis testing, the test statistic (e.g. *F*, *t*, *r*) with confidence intervals, effect sizes, degrees of freedom and *P* value noted<br>*Give P values as exact values whenever suitable.*
☒	☐	For Bayesian analysis, information on the choice of priors and Markov chain Monte Carlo settings
☒	☐	For hierarchical and complex designs, identification of the appropriate level for tests and full reporting of outcomes
☒	☐	Estimates of effect sizes (e.g. Cohen's *d*, Pearson's *r*), indicating how they were calculated

Our web collection on statistics for biologists contains articles on many of the points above.

Software and code

Policy information about availability of computer code

Data collection	Intracellular electrophysiological data were digitized with a Digidata 1440 (Molecular Devices) A/D board and recorded as voltages (current clamp recordings) or currents (voltage clamp recordings) with the Clampex module of pClamp10.7 software (Molecular Devices). Extracellular recordings were acquired with a Digidata 1550B (Molecular Devices) and recorded with the Axoscope module of pClamp10.7 software. High speed video was acquired with a Photron FASTCAM Mini UX100 camera and recorded with Photron FASTCAM viewer sofware (PFC version 3.6.9.0). Calcium imaging was conducted with a Hamamatsu CMOS camera (model C11440-42U) mounted to a Zeiss Axioscope 2FS fluorescence microscope. Confocal imaging was conducted with a Leica TCS SP8 laser scanning microscope with a HC PL APO 40x oil lens (NA 1.3) and images were acquired with Leica Application Software (LasX, 3.5.2.18963).
Data analysis	All extracellular electrophysiological data were pre-processed with the Clampfit module of pClamp10.7 software and imported into Spike2 (version 7.2) software (Cambride Electronic Design). Analyses were done with Spike2 (version 7.2) and custom written Python routines (Jupyter Notebook with the NumPy, pickle, SciPy and Matplotlib Python libraries). Intracellular recordings were analyzed with the Clampfit module of pClamp10.7 software. Confocal imaging data were analyzed with AMIRA 4.1 (Thermo Fisher) and Huygens Professional software (version 19.10.0). Statistical analyses were conducted with SPSS Statistics 22 (IBM) and Graphpad Prism 9.2.0 (GraphPad Software, San Diego).

For manuscripts utilizing custom algorithms or software that are central to the research but not yet described in published literature, software must be made available to editors and reviewers. We strongly encourage code deposition in a community repository (e.g. GitHub). See the Nature Portfolio guidelines for submitting code & software for further information.

Data

Policy information about availability of data

All manuscripts must include a data availability statement. This statement should provide the following information, where applicable:

- Accession codes, unique identifiers, or web links for publicly available datasets
- A description of any restrictions on data availability
- For clinical datasets or third party data, please ensure that the statement adheres to our policy

Data availability: The electrophysiological and imaging data have been deposited to the Zenodo open data repository and are available at doi: 10.5281/zenodo.7737730. Computational modelling data and additional computational analyses of in vivo electrophysiological data have been deposited to the Zenodo open data repository and are available at doi: 10.5281/zenodo.7740678.

Code availability: Code for implementing the computational model and for electrophysiological data analyses is available at doi: 10.5281/zenodo.7740678.

Human research participants

Policy information about studies involving human research participants and Sex and Gender in Research.

Reporting on sex and gender	n/a
Population characteristics	n/a
Recruitment	n/a
Ethics oversight	n/a

Note that full information on the approval of the study protocol must also be provided in the manuscript.

Field-specific reporting

Please select the one below that is the best fit for your research. If you are not sure, read the appropriate sections before making your selection.

☒ Life sciences ☐ Behavioural & social sciences ☐ Ecological, evolutionary & environmental sciences

For a reference copy of the document with all sections, see nature.com/documents/nr-reporting-summary-flat.pdf

Life sciences study design

All studies must disclose on these points even when the disclosure is negative.

Sample size	No statistical tests were used to determine sample size. We used sample sizes (7-100 flies per condition) that have previously been shown to have sufficient statistical power in similar experiments in the past (e.g. Ryglewski et al., 2014, 2017; Krick et al., 2021).
Data exclusions	We did not exclude samples from our analyses unless animals were unable to perform the behavioral task. Patch Clamp recordings were discarded if technical quality criteria (access resistance above 12MO and motoneuron input resistance below 100MO) were not met.
Replication	Figure 1A (traces). Representative example traces of extracellular recording of DLM-motoneurons MNs1-5 (8 replicates in 8 different animals).<br>Figure 1B: 8 replicates in 8 different animals (animals were excluded only if they were unable to perform the task).<br>Figure 1C: red dots, 100 different animal (animals were excluded only if they were unable to perform the task); gray dots, value pairs from one representative animal with a range of MN firing frequencies (4 replicate animals).<br>Figure 1D: Representative current clamp traces from one animal (15 replicate animals). Recordings were excluded only if quality criteria were not met (quality criteria for all patch clamp recordings were input resistance >100MO and access resistance <12MO).<br>Figure 1E: Data from 15 replicate animals. Recordings were excluded only if quality criteria were not met.<br>Figure 1F: Data from same 8 animals as Figure 1B.<br>Figure 1G: representative spike time events from 1 animal (8 replicate animals).<br>Figure 2A: Representative traces of extracellular MN4 and MN5 recordings from 1 animal per genotype. 10 replicate animals for each genotype.<br>Figure 2B: Data from 10 animals per genotype. Recordings were excluded only if animals did not perform the flight task.<br>Figure 2C: One representative image from 3 replicate animals.<br>Figures 2D, E: Representative paired recordings from 6 replicate animals.<br>Figure 2F: Analyses from 16 paired recordings from 10 animals.<br>Figure 2G: Representative paired recording with ShakB RNAi knock-down (3 replicate animals).<br>Figures 2H, I: Representative paired recording (3 replicate animals).<br>Figure 3B: Representative network simulation with electrical coupling coeffcient of 0.005 (10 simulations for this condition).<br>Figure 3C: Computational modelling with 200 simulations per coupling coefficient.<br>Figure 3D: Simulations were run 10 times per condition with the final parameter sets. No simulations were excluded. Experimental data

included 9 replicates from different animals for Shak-B-RNAi, 7 replicates from different animals for Shak-B overexpression, and 11 replicate control animals. We confirmed that there were no apparent differences between the MN4+5 synchronisation index of animals with only MN4 and MN5 recorded (7 animals) and the MN4+5 synchronisation index of those animals with all 5 units recorded (4 animals). No animals were excluded.
Figure 3F: Represenative voltage clamp recordings with 6 replicate control animals and 5 replicate animal with Shab-overexpression.
Figure 3G: Representative extracellular rcordings from 10 replicate animals for Shab overexpression and 8 replicate control animals.
Figure 3H: Simulations were run 10 times per condition with the final parameter sets. No simulations were excluded. In vivo data with 10 replicate animals for Shab overexpression and 8 replicate control animals. No animals were excluded.
Figure 3J: Analyses of computational model (10 simulations per condition with the final parameter set, so simulations excluded) and in vivo data with 8 replicate animals. In vivo data was excluded only if animals were unable to perform the task.
Figure 4A: Representative traces from 1 animal (7 replicate animals).
Figure 4B: top, representative traces from one animal (3 replicate animals); middle, representative trace from 1 animal (7 replicate animals); bottom, representative trace from 1 animal (7 replicate animals).
Figure 4C: Represenative calcium imaging traces (3 replicate animals).
Figure 4E: average traces from 7 replicate animals per condition.

Randomization

Different experimental groups are defined by genotype in this study. Data acquisition from different genotypes was fully randomized.

Blinding

For all in vivo recordings the experimenter was blind to genotype (the type of genetic manipulation or control). In immunohistochemical experiments blinding was not required because no comparisons between experimental groups were conducted. All data analyses for computational modeling was fully automated with no data excluded so that blinding was not required.

Reporting for specific materials, systems and methods

We require information from authors about some types of materials, experimental systems and methods used in many studies. Here, indicate whether each material, system or method listed is relevant to your study. If you are not sure if a list item applies to your research, read the appropriate section before selecting a response.

Materials & experimental systems

n/a	Involved in the study	
☐	☒	Antibodies
☒	☐	Eukaryotic cell lines
☒	☐	Palaeontology and archaeology
☐	☒	Animals and other organisms
☒	☐	Clinical data
☒	☐	Dual use research of concern

Methods

n/a	Involved in the study	
☒	☐	ChIP-seq
☒	☐	Flow cytometry
☒	☐	MRI-based neuroimaging

Antibodies

Antibodies used

1. Chicken Anti-GFP primary antibody, polyclonal (Thermo Fisher, Cat # A10262)
2. Rat anti-mCherry monoclonal primary Antibody (clone 16D7) (Thermo Fisher, Cat # M11217)
3. Secondary antibody donkey anti-chicken Alexa Fluor 488 (Jackson Immunoresearch, Cat # 703-545-155)
4. Secondary antibody donkey anti-rat Alexa Fluor 594 (Jackson Immunoresearch, Cat # 712-587-003)

Validation

1. AB specificity verified by Thermo Fisher. Antibody was verified by relative expression to ensure that the antibody binds to the antigen stated (https://www.thermofisher.com/antibody/product/GFP-Antibody-Polyclonal/A10262).
2. AB specificity verified by Thermo Fisher in immunohistochemistry with mCherry transfected cells (https://www.thermofisher.com/antibody/product/mCherry-Antibody-clone-16D7-Monoclonal/M11217).
3. AB specificity verified based on immunoelectrophoresis and/or ELISA by Jackson Immunoresearch (https://www.jacksonimmuno.com/catalog/products/703-545-155).
4. AB specificity verified based on antigen binding test and/or ELISA by Jackson Immunoresearch (https://www.jacksonimmuno.com/catalog/products/712-587-003).

Animals and other research organisms

Policy information about studies involving animals; ARRIVE guidelines recommended for reporting animal research, and Sex and Gender in Research

Laboratory animals

All Drosophila melanogaster flies were 2-5 days of age. The following Drosophila melanogaster fly strains were used.
1. DLM-Split GAL4: w; P{GMR23H06-pBPp65ADZpUw}attP40; P{GMR30A07-pBPZpGAL4.BD.Uw}attP2
2. DLM-GAL4: w;+; P{GMR23H06-GAL4} attP2
3. DLM-Split GAL4 with CD4-tdGFP expression: w; P{GMR23H06-pBPp65ADZpUw}attP40 P{UAS-CD4-tdGFP}8M2; P{GMR30A07-pBPZpGAL4.BD.Uw}attP2
4. GAL4 expression in DLM indirect flight muscle: w;+;P{Act88F-GAL4.1.3}3
5. GAL4 expression in period-interneurons: w;P{w[+mC] = GAL4-per.BS}3;+
6. LC4 visual interneuron Split GAL4: w; P{y[+t7.7]w[+mC] = R47H03-p65.AD}attP40; P{y[+7.7]w[+mC] = R86D05-GAL4.DBD}attP2

7. DLM-Split GAL4 with expression of CD4-tdTomato: w; P{GMR23H06-pBPp65ADZpUw}attP40 P{UAS-CD4-tdTom}7M1; P{GMR30A07-pBPZpGAL4.BD.Uw}attP2
8. UAS-RNAi of ShakB innexin: y[1]sc[*]v[1]sev[21]; +; P{TRiP.HMC04895}attP2
9. UAS-transgene of ShakB N+16: w;UAS-shakB(N+16);+
10. Control for mini-white based transgenic insertions: w[1118] or w*
11. Control for VALIUM-based transgenic insertions: y[1]v[1];+;P{UAS-GFP.VALIUM}attP2
12. UAS-RNAi transgene for GluClα: w[1118]+;P{KK109167}VIE-260B
13. UAS-RNAi transgene for GABAA Rdl receptor: w[1118]+;P{GD4609}v41103
14. UAS-transgene for the expression of Channelrhodopsin2.XXL: w[1118];P{UAS-ChR2.XXL}VK00018
15. UAS-RNAi transgene for FMRP fragile X mental retardation protein: w[1118]+;P{KK107935}VIE-260B
16. UAS-transgene for expression of GCaMP8f: w;P{20XUAS-IVS-jGCaMP8f}su(Hw)attP5;+
17. trans-tango: w* P{UAS-myrGFP.QUAS-mtdTomato-3xHA};P{y[+7.7]w[+mC] = trans-Tango}attP40;+
18. UAS-transgene to express a red-fluorescent truncated version of the synaptic marker bruchpilot: w*; UAS-brp.S(D3)-mStrawberry;+
19. UAS-Shab: w*; P{UAS-Shab}pJFRC81 attP2
20. UAS-RNAi of Shab: y[1]sc[*]v[1]sev[21]; P{UAS-TRiP.HMC04895}attP40;+
21. activity dependent GRASP: w*; P{w[+mC] = lexAop-nSyb-spGFP1-10}2, P{w[+mC] = UAS-CD4-spGFP11}2; TI{2A-lexA::GAD}ChAT[2A-lexA]
22. wildtype strain Canton Special: +;+;+;+

Apis mellifera were collected at ages 4-6 weeks from the local apiculture of the Johannes Gutenberg University Mainz. Other insect species (Calliphora spec., Drosophila hydei, Lucilla spec., Musca domestica) were obtained from local pet shops as feed insects.

Wild animals	No wild animals were used in this study
Reporting on sex	Only male animals were used in this study.
Field-collected samples	No animals were collected in the wild
Ethics oversight	No ethical oversight was required for work with insect species. The work was according to the guidelines of research with transgenic invertebrate animals of the state of Rhineland-Palatinate in Germany.

Note that full information on the approval of the study protocol must also be provided in the manuscript.
